# The Role of Proton Magnetic Resonance Spectroscopy in Neonatal and Fetal Brain Research

**DOI:** 10.1002/jmri.29709

**Published:** 2025-01-21

**Authors:** Steve C.N. Hui, Nickie Andescavage, Catherine Limperopoulos

**Affiliations:** ^1^ Developing Brain Institute, Children's National Hospital Washington D.C. USA; ^2^ Department of Radiology The George Washington University School of Medicine and Health Sciences Washington D.C. USA; ^3^ Department of Pediatrics The George Washington University School of Medicine and Health Sciences Washington D.C. USA; ^4^ Division of Neonatology Children's National Hospital Washington D.C. USA; ^5^ Prenatal Pediatric Institute, Children's National Hospital Washington D.C. USA

**Keywords:** brain, fetuses, metabolites, neonates, proton magnetic resonance spectroscopy

## Abstract

The biochemical composition and structure of the brain are in a rapid change during the exuberant stage of fetal and neonatal development. ^1^H‐MRS is a noninvasive tool that can evaluate brain metabolites in healthy fetuses and infants as well as those with neurological diseases. This review aims to provide readers with an understanding of 1) the basic principles and technical considerations relevant to ^1^H‐MRS in the fetal‐neonatal brain and 2) the role of ^1^H‐MRS in early fetal‐neonatal development brain research. We performed a PubMed search to identify original studies using ^1^H‐MRS in neonates and fetuses to establish the clinical applications of ^1^H‐MRS. The eligible studies for this review included original research with ^1^H‐MRS applications to the fetal‐neonatal brain in healthy and high‐risk conditions. We ran our search between 2000 and 2023, then added in several high‐impact landmark publications from the 1990s. A total of 366 results appeared. After, we excluded original studies that did not include fetuses or neonates, non‐proton MRS and non‐neurological studies. Eventually, 110 studies were included in this literature review. Overall, the function of ^1^H‐MRS in healthy fetal‐neonatal brain studies focuses on measuring the change of metabolite concentrations during neurodevelopment and the physical properties of the metabolites such as *T*_1_/*T*_2_ relaxation times. For high‐risk neonates, studies in very low birth weight preterm infants and full‐term neonates with hypoxic–ischemic encephalopathy, along with examining the associations between brain biochemistry and cognitive neurodevelopment are most common. Additional high‐risk conditions included infants with congenital heart disease or metabolic diseases, as well as fetuses of pregnant women with hypertensive disorders were of specific interest to researchers using ^1^H‐MRS.

**Evidence Level:**

1

**Technical Efficacy:**

Stage 2

Magnetic resonance spectroscopy (MRS) is a noninvasive imaging tool that is particularly useful for monitoring brain biochemistry in pathological conditions. MRS can provide valuable information on neurochemical changes that occur in healthy newborns, those at risk for brain injury, or for evaluation of neurological disorders. However, MRS studies can be challenging from an acquisition and quantification perspective in the fetal and neonatal period, given the rapid developmental changes in the immature developing brain. This 2‐part review sets out to 1) describe the basic principles and technical considerations of MRS in fetal‐neonatal neuroimaging and 2) provide readers with a comprehensive overview of the role and application of ^1^H‐MRS in the study of the fetal‐neonatal brain with an emphasis on future directions that improve its accuracy, reliability, and consistency for data acquisition in both research and clinical situations.

## Basic Principle

MRS apply the same basic principle as magnetic resonance imaging (MRI) to utilize MR scanners to detect radiofrequency (RF) signals from nuclei possessing nuclear spin. The signals arising from nuclei spin in different local chemical environments are separated along the frequency axis due to the difference in the resonant frequency known as chemical shift. For in vivo MRS, the most observed nucleus is the hydrogen proton (^1^H), followed by carbon (^13^C), phosphorus (^31^P), sodium (^23^Na), and xenon (^129^Xe).^1^, ^2^ Due to a proton's high gyromagnetic ratio, high natural abundance and presence in most molecules in the body, ^1^H‐MRS is the most popular modality for studying neuro‐metabolite concentrations in clinical applications. It detects molecules other than water characterized by their distinct chemical shifts for metabolite modeling. It has been commonly used to detect endogenous tissue metabolites. The minimum detectable metabolite concentration is in the order of 0.5–1 mM.^3^, ^4^, ^5^ The area under the resonance peak is proportional to the number of protons of different resonances within one molecule and to concentrations of the metabolites. Other contributing factors including *J*‐coupling and echo time (TE) also impact the overall peak areas. Since most in vivo metabolites contain protons, the high sensitivity of ^1^H‐MRS induces an inherent limitation of overlapping metabolite resonances in a narrow chemical shift range, especially for upfield metabolites. The considerable number of overlapping metabolite resonances can make the quantification challenging at clinical field strength.

## Quantification

**FIGURE 1:**
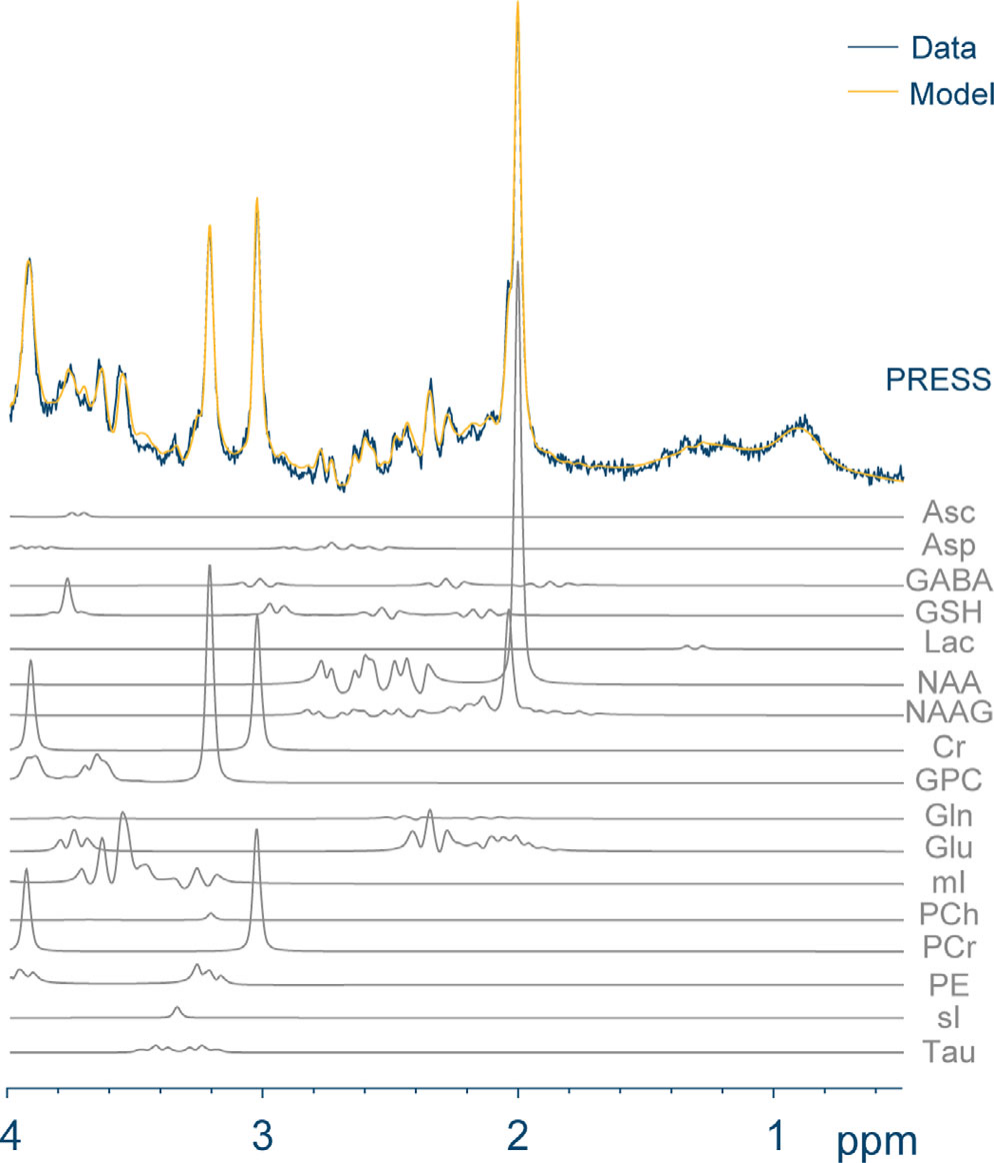
A representative basis set applied to model spectral data acquired from a healthy neonate using Osprey. Seventeen metabolite basis functions were included and presented. Linear combination modeling was performed to model the in vivo spectrum (blue) and the resulting fits were shown (yellow). Asc = ascorbic acid; Asp = aspartic acid; GABA = gamma‐aminobutyric acid; GSH = glutathione; Lac = lactate; NAA = N‐acetyl aspartate; NAAG = N‐acetyl aspartyl glutamate; Cr = creatine; GPC = glycerophosphocholine; Gln = glutamine; Glu = glutamate; mI = myo‐inositol; PCh = choline‐containing compounds; PCr = phosphocreatine; PE = phosphorylethanolamine; sI = scyllo‐inositol; Tau = taurine.

Following data acquisition, spectral quantification is the next most critical step. It is performed directly after preprocessing are complete. To estimate the spectral peak areas of different metabolites, existing methods such as linear combination modeling (LCM), peak fitting, and peak integration have been implemented in many popular fitting algorithms including LCModel,^6^ Tarquin,^7^ FSL‐MRS,^8^ and Osprey.^9^ A community consensus has recently coalesced and has recommended the use of LCM fitting to quantify metabolite spectra^10^ due to its advantage in fitting each metabolite as an individual function called a basis spectrum. In LCM, the acquired spectrum is modeled as a weighted sum of metabolite basis functions (Fig. 1), and the basis set can be generated either by a set of single‐metabolite phantoms or by numerical simulation.^11^, ^12^ Common metabolites of interest will be introduced in the following section.

## Metabolites of Interest

Quantitative ^1^H‐MRS has a broad range of preclinical and clinical research applications. In the brain, changes in metabolite levels can be observed in neurodevelopment, neurodegeneration, neurological diseases, or psychiatric disorders.^13^ ^1^H‐MRS also can be a potential tool to detect biomarkers for brain tumors although the prevalence is very low in neonates.^14^, ^15^, ^16^ Metabolites of interest with high concentrations including N‐acetyl aspartate (NAA), creatine (Cr), myo‐inositol (mI), glutamine (Gln), and glutamate (Glu) (collectively as Glx), and choline (Cho) can be observed and quantified from spectra acquired using unedited short‐TE localization sequences.^17^ NAA, Cr, and Cho have been the most common and conventional metabolites of interest to be investigated since the beginning of in vivo research to study brain biochemistry using ^1^H‐MRS. NAA is an important biomarker for neuronal health and neurodevelopment. It has been often used as an indicator of neuro fiber integrity. Cho has the highest level of concentration during the prenatal, decreases rapidly during early neurodevelopmental stage and becomes stable after birth.^18^ Some evidence shows that an increased level of Cho exerts neuroprotective effects to the injured brain and improve brain recovery.^19^ Cr is often used to monitor altered energy metabolism as it is a central energy marker and responsible for energy storage and transfer. It is relatively stable in most conditions, so it has been used as an internal reference for comparison to other metabolites.^20^ Some metabolites are presented at detectable levels (of the order of 1 mM) but cannot be resolved due to the limited chemical shift dispersion and overlying signals of more concentrated metabolites at similar resonance frequencies. *J*‐difference editing is the most common approach for spectral editing which has been widely implemented in many editing schemes.^21^, ^22^, ^23^, ^24^, ^25^ These editing schemes distinguish resonances of low‐concentration metabolites from overlying signals. γ‐aminobutyric acid (GABA) is one of the most popular metabolites being studied using these editing schemes.^26^ It serves as the main inhibitory neurotransmitter in the mature brain. Noteworthy for this current review, it is an excitatory neurotransmitter in the fetus and newborn.^27^ Other metabolites of target in edited MRS included glutathione (GSH, another excitatory neurotransmitter besides GABA and Glu),^28^ ascorbic acid (Asc, also known as vitamin C),^29^ aspartic acid (Asp), lactate (Lac, can also be measured using unedited MRS at TE 144 or 288 msec),^30^ phosphorylethanolamine (PE),^31^ ethanol (EtOH, an exogenous compound),^32^ 2‐Hydroxyglutarate (2HG, a biomarker for a subset of gliomas)^14^ etc. Characteristics of each individual and clinically relevant compound have been previously described in detail.^33^, ^34^

## Technical Challenges

MRS comes with technical challenges, and the wider research community has been working to improve the overall quality of the data acquisition extensively. Early MRS consensus papers have described issues and potential solutions that improve acquisition, shimming and processing to tackle artifacts in data acquired from the general population.^35^ In fetal and neonatal ^1^H‐MRS, there are the added difficulties associated with involuntary motions, poor shimming due to the smaller brain volume and the dynamic change of the brain structure and unknown *T*_1_/*T*_2_ relaxation times in brain tissues and metabolites as obstacles that must be addressed to maintain the data quality.

### Motion

**FIGURE 2:**
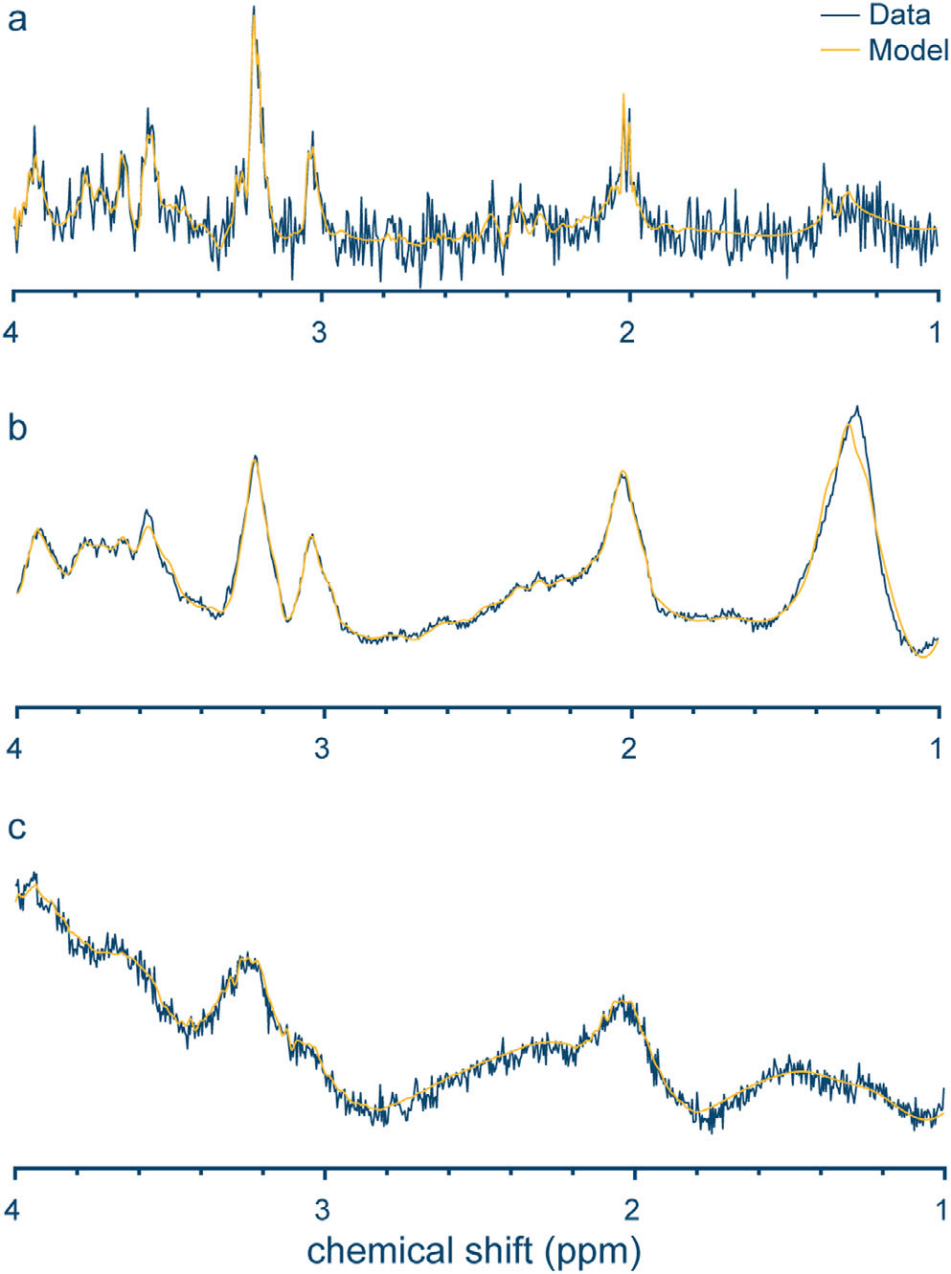
A representative in vivo spectrum with (**a**) low signal‐to‐noise ratio, (**b**) lipid contamination, and (**c**) bad shimming. Data were acquired in the right frontal lobe of neonates using PRESS 3 T (TE/TR: 35 msec/1500 msec; transients: 128).

Motion is one of the most challenging issues to overcome in fetal‐neonatal MRS and it can have a pronounced effect that inevitably degrades the quality of data acquisition. Spectra with severe motion will result in spectral distortions, increased linewidths, lower signal‐to‐noise ratio (SNR; Fig. 2a), unwanted sampling of tissue outside the region of interest (eg, lipid contamination from the skull due to head movements; Fig. 2b), and incoherent averaging and subtraction artifacts due to chemical shifts. This is particularly important for neonatal and fetal scans performed in a non‐sedated environment because involuntary head motions happen frequently during natural sleep. Current consensus has recommended three common approaches that can be employed for mitigating motion, including 1) subject immobilization, 2) retrospective correction, and 3) prospective real‐time correction using tracking methods. Visual inspection or automatic outlier detection algorithm can be applied to remove corrupted transients. Prospective corrections including acquisition of rapid navigator images between repetitions and optical tracking can precisely monitor subject motion to update the acquisition volume in real‐time.^36^ Some studies in the older pediatric population applied image‐based navigators to mitigate motion artifacts on sick children during MRS acquisitions.^37^, ^38^ However, many other consensus recommendations that deal with motion artifacts have not been applied on neonatal and fetal scans.

### Shimming

Shimming or *B*_0_ field homogenization reduces field spatial variation using shim coils. It has been a common issue for MRI and MRS. However, MRS is particularly susceptible to bad shimming (Fig. 2c) because it relies on precise measurements of metabolite peaks within a small voxel especially in the developing and irregular brain shape of infants. Impact of *B*_0_ inhomogeneity on localization accuracy leading to incorrect slice profile and spatial position for voxel localization that reduces SNR, broadens spectral width and induces spectral distortions.^39^ Real‐time higher‐order (>first) shim provides greater ability to correct the complex variations due to the increase of spatial complexity with higher order in the linear combination of spherical harmonic functions. In neonatal and fetal scans, it is particularly difficult to achieve a good shim due to the relatively small brain volume and the non‐uniform shape of the targeted brain regions. In addition, fetal scans are further complicated by the spatial location which further deteriorates the shim given the distance of fetal brain tissue relative to coil placement and additional layers of intervening tissue (amniotic fluid, placenta, maternal uterus, and subcutaneous tissues). Repeated or manual shimming and repositioning of the mother may improve the shim quality. However, the effort does not always lead to success.

### *T*_1_ / *T*_2_ Relaxation

**FIGURE 3:**
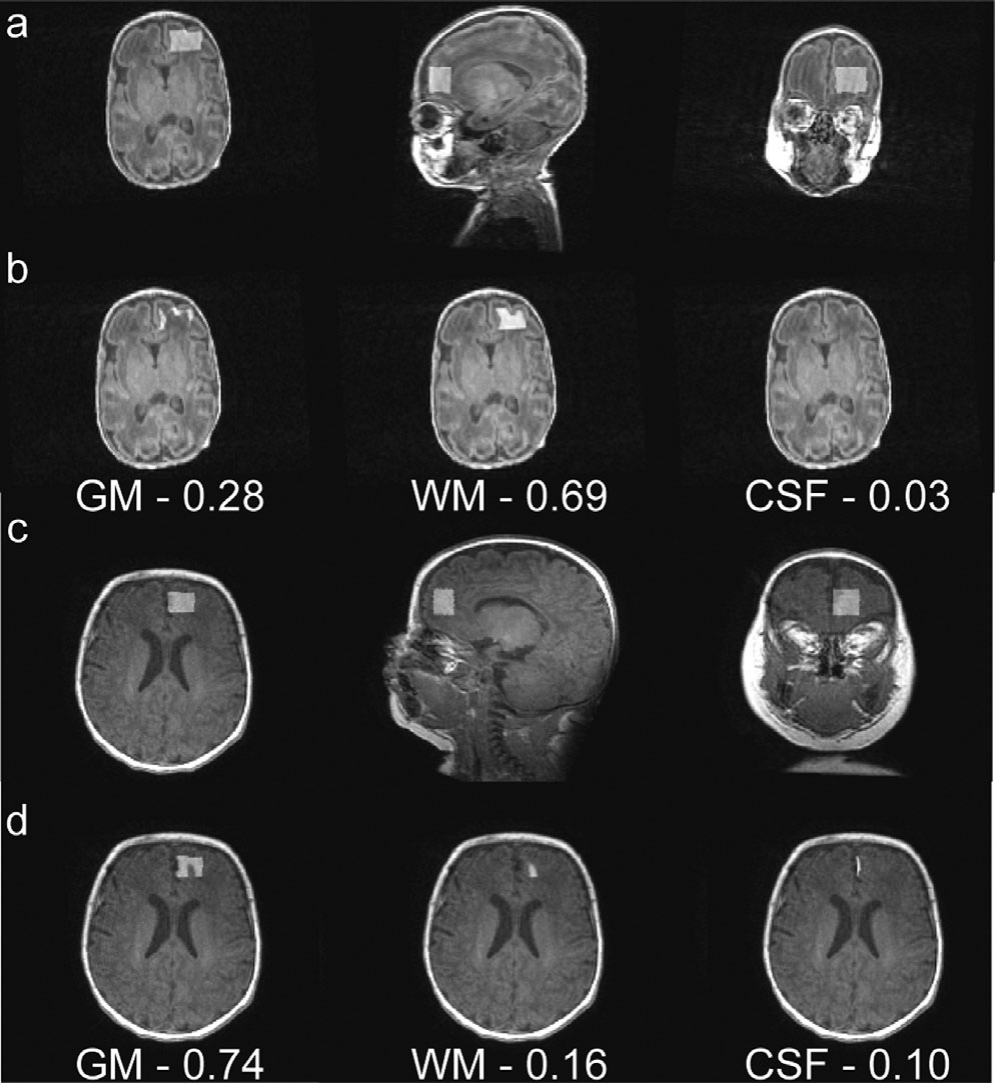
*T*_1_‐weighted brain images of (**a**) a preterm neonate (female, GA at MRI—32 weeks) and (**c**) a healthy term neonate (female, GA at MRI—45 weeks). Panels (**b**) and (**d**) are the segmentations of the MRS voxels. As shown in panel (d), contrast between white matter and gray matter is less differentiated as myelinated white matter becomes brighter compared to unmyelinated white matter in panel (b), resulting 16% white matter segmentation in the white‐matter‐rich right frontal lobe compared to 69% in panel (b) in which the myelination process has been initiated. The rapid changes of *T*_1_/*T*_2_ relaxation times due to the dynamic progression of myelination that induces the lack of contrast between white matter, gray matter and cerebrospinal fluid may led to inaccurate tissue segmentations within the MRS voxel and *T*_1_/*T*_2_ correction for quantification of metabolite concentrations.

In fetuses and neonates, the *T*_1_ and *T*_2_ relaxation times of white matter and gray matter rapidly change due to the dynamic progression of myelination.^40^ *T*_1_ relaxation of white matter increases relative to gray matter and vice versa for *T*_2_ relaxation between the second and third trimester of gestation through the first months of life for infants^41^ (Fig. 3). Due to the rapid change of *T*_1_ and *T*_2_ relaxation times, brain regions and tissue segmentations can be challenging in fetuses and neonates. Brain templates generated using the fetal and neonatal populations allow more accurate brain region segmentations.^42^, ^43^ For ^1^H‐MRS, metabolite measurements are impacted by some degree of variation due to the tissue compositions of the voxel (i.e., the amount of white matter, gray matter, and cerebrospinal fluid within the MRS voxel). An accurate tissue segmentation within the voxel and the correct values of *T*_1_/*T*_2_ relaxation times of the tissues for fetus and neonate allow tissue corrections that account for the metabolite contribution from different tissue types.^44^

To quantify using LCM, metabolite *T*_1_/*T*_2_ relaxation times can be applied to correct any signal loss during decay. These values were reported in early studies using multiple TEs and TRs in healthy and preterm infants. *T*_2_ relaxation times were higher in NAA, Cr, Cho, and mI in neonates when compared to the adult populations; *T*_1_ relaxation had a similar regressive effect on Cr and mI as a function of age in infants. The inverse was true for NAA and Cho. For preterm neonates, *T*_1_/*T*_2_ relaxation times were reported in postconceptional age of 37.8 weeks in which results were comparable but noticeably different from those in healthy neonates^45^, ^46^, ^47^ (Table 1). However, in most LCM tools, *T*_1_ relaxation times are assumed to be constants across age and *T*_2_ relaxation corrections are applied using values obtained from adult populations. These data suggest that those assumptions may not be valid during the early developmental stages, nor across disease states.^48^, ^49^, ^50^

**TABLE 1:** Clinical Data and Protocol for Studies in *T*_1_/*T*_2_ Relaxation Times (msec) for Healthy Preterm and Term Neonates

	N	Brain Regions	TR (msec)	TE (msec)	Field Strength	Vendor	Sequence	Major Findings (msec)
Kreis et al 1993	77	Mid. and parieto‐occipital cortex	1500/3000/5000	30/40/60/90/135/270	1.5 T	GE	STEAM	NAA/Cr/Cho/mI: *T*_1_: 930/1620/1320/1520 (higher Cr/mI and lower NAA/Cho in infants compared with adults) *T*_2_: 524/228/431/301 (all higher in infants compared with adults)
Toft et al 1994	22	Striatum	1600/6000	20/46/92/272	1.5 T	Siemens	STEAM	NAA/tCr/Cho/Inositol: *T*_2_: 324/216/398/76
Kugel et al 2003	84	Basal ganglia	1884/2000/6000	25/136/272	1.5 T	Philips	PRESS	NAA/Cr/Cho/mI/Lac: *T*_1_: 1171/1388/1217/1336/1820 *T*_2_: 499/224/273/68/1022

### Frequency and Phase Drifts

In fetal‐neonatal scans, random translational head motion leads to extra phase shifts^51^ in addition to the impact of frequency drifts that deteriorate editing efficiency and co‐editing contributions which ultimately induce subtraction artifacts. The overall spectral quality reduces if no correction is being made especially for edited MRS. Prospective real‐time movement and frequency corrections mainly apply in MRS of adult population are rare in pediatric studies. Future application of them in fetal‐neonatal scans would improve data acquisition by minimizing the impact from frequency and phase drifts.

## Methods

^1^H‐MRS has frequently aided in the investigation of early fetal‐neonatal brain development. Previous reviews in neonates and children cover topics for unique metabolites,^52^ specific MRS modalities,^53^ editing sequences,^34^ and older pediatric populations.^54^, ^55^ While MRS technologies improve, new tools and new sequences emerge leading to new implementations, improvements, and applications that previous literature may not have covered. To keep members of the MRS community up to date, there is a need to renew our knowledge and give an update to the previous review literature based on long‐standing established research.^54^, ^56^

### Searching Methods

The literature review in PubMed used the following keywords: proton magnetic resonance spectroscopy neonate OR proton magnetic resonance spectroscopy fetus NOT review NOT meta‐analysis NOT books NOT documents. The period for publication included in this review was the year 2000 to the year 2023. This was to keep the content and discussion of the manuscript relevant. This review also accounts for landmark publications in the 1990s that made significant findings from the early phase of neonatal MRS usage for clinical applications. These landmark publications were selected for their impact (citations >100) relevant to healthy term or preterm neonates, neurodevelopment, neurological diseases, cardiopulmonary, and metabolite conditions. A separate section of fetal MRS had been included to summarize emerging data on the role of MRS in the developing brain.

### Exclusion and Inclusion

**FIGURE 4:**
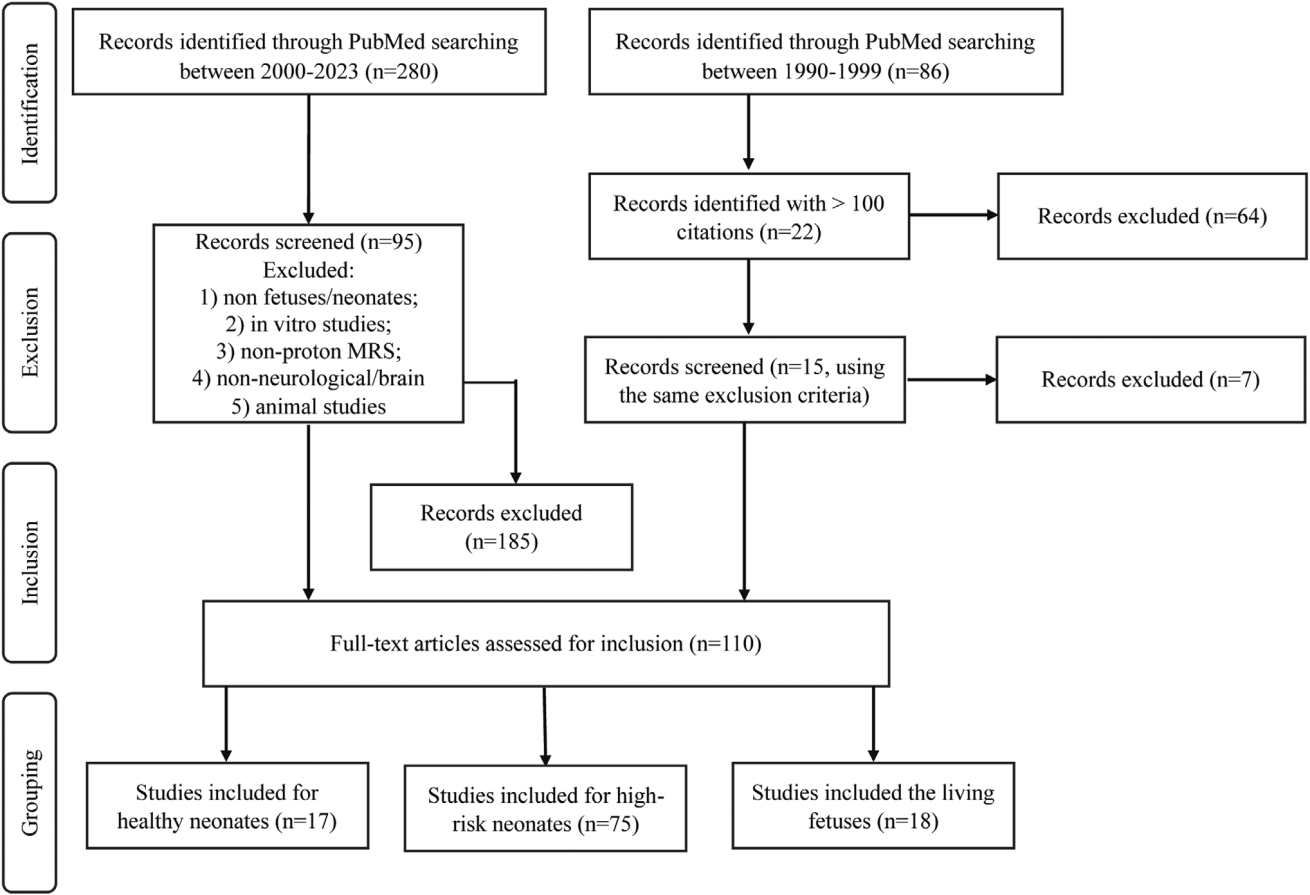
A flowchart for the steps of searching relevant literature from identification to exclusion and inclusion followed by grouping for analysis.

PubMed provided 366 results (280 publications between 2000 and 2023 and 86 between 1990 and 1999) using key words mentioned previously. After excluding original studies that did not involve any fetuses or neonate groups within the first 4 weeks of life; in vitro, non‐proton MRS, non‐neurological/brain, and animal studies were also excluded. Ninety‐five papers between 2000 and 2023 and 15 landmark papers between 1990 and 1999 were included in this literature review. They were categorized and presented in three main groups: 1) MRS in healthy neonates, 2) MRS on neonates with neurological disease and illnesses, and 3) MRS on living fetuses. The steps used to identify, include, and exclude papers for this review are presented in a flowchart (Fig. 4). The following sections provide its readers with a comprehensive overview of ^1^H‐MRS for recent research and clinical applications in the fetal‐neonatal developing brain.

## MRS in Healthy Neonates

### Dynamic Change of Brain Metabolites Concentration During Early Neurodevelopment

One of the most predominant roles of fetal‐neonatal ^1^H‐MRS is to study the dynamic change of brain metabolites in the developing brain during the early stage of infancy. NAA is one of the most abundant metabolites in the human brain.^54^ Several studies showed a positive correlation of NAA concentrations to the increasing gestational age of neonates in the cerebellum, frontal lobe, basal ganglia, and thalamus.^46^, ^57^, ^58^, ^59^, ^60^, ^61^, ^62^ Since NAA is an important biomarker for neuronal health and neurodevelopment during infancy, measurements of NAA using ^1^H‐MRS have been used as indicator for healthy brain maturation. For other metabolites, two studies reported on Tau as it increased in cerebellum^57^ and decreased in white matter of centrum semiovale along with brain maturation.^63^ Two studies reported an increase of tCr and a decrease of inositol/Cr as a function of advancing gestational age^46^, ^58^; reduced Glx was reported in centrum semiovale in preterm neonates compared with full‐term neonates.^63^ These results generated the brain biochemical profile in healthy neonates during their early stage of neurodevelopment and provided references for metabolite comparisons between healthy and sick neonates with neurological diseases or brain lesions.

Recently, advanced edited sequences^23^, ^24^, ^25^ were implemented to study low‐concentrated metabolites such as GABA in addition to the conventional high concentration metabolites as mentioned. A longitudinal study reported common brain metabolites as well as GABA from the right basal ganglia in a group of preterm neonates at 37–46 and 64–73 postmenstrual weeks.^64^ GABA measurements were contradicted based on the reporting method. GABA/Cr ratio decreased significantly as a function of age but not in GABA/Cho ratio and water‐scaled GABA measurement. A similar contradiction was found in a cross‐sectional study. Water‐scaled GABA+ (i.e., GABA+ macromolecules) of infants in the basal ganglia and cerebellum were significantly lower than those in children. Contradictory results were reported for measurements in Cr ratios as preterm and term infants had a significantly higher GABA+/Cr ratio in the cerebellum compared with children.^65^ The discrepancy between water‐scaled and Cr ratios measurement may be the result of the differences of Cr concentrations, due to the changes in the early stage of neurodevelopment in the basal ganglia and cerebellum between newborns and children, in which the inconsistent denominator dominated the calculation. Reporting methods must be used carefully especially for fetal‐neonatal measurements because metabolites that are thought to be stable could change rapidly over the early stage of neurodevelopment. Results could be biased for longitudinal studies that reported their measurements in metabolite ratios due to the significant changes in the denominator of the calculations. Besides GABA, a systemic approach to measure low concentration metabolites including GSH and Glx in the neonatal population has been investigated using the advanced Hadamard encoding and reconstruction scheme.^66^ However, the application of the advanced editing sequences has been very limited in fetal‐neonatal studies given its ability to measure many important in vivo brain molecules including but not limited to GABA, GSH, Glx (Glu + Gln), Lac, Asc, Asp etc.

### Regional Differences in Brain Metabolites

Numerous studies to date have reported on regional brain metabolite concentrations, particularly in the cerebellum, basal ganglia, and gray/white matter of the frontal lobe. One study reported regional differences in brain metabolite measurements between healthy and preterm newborns.^67^ The overall NAA, Cho, and Cr concentrations were significantly higher in the cerebellum in term infants compared to preterm infants after adjustments for postmenstrual age. Glx/Cho ratio in the right basal ganglia, GABA+, Cho, and GABA+/Cho ratio in the right frontal lobe were overall increased in term neonates compared with preterm neonates.^67^ In the cerebellum, there was a relative increase in GABA+, Glx, Cho, Glu, Gln, GSH, and myo‐inositol compared to concurrent measures in the basal ganglia. Conversely, the basal ganglia had higher levels of NAA and Cr. In terms of Cho ratios, GABA+, Glx, NAA, and Cr were highest in the basal ganglia.^60^ For more advanced editing schemes, Hadamard‐encoded MRS on healthy neonates was first reported in 18 term‐born neonates (median postmenopausal age: 41 weeks, range 39–47 weeks) to study GABA+, GSH, and Glx.^66^ When a *T*_2_ correction was applied to the measurements, GABA+/water and Glx/water ratios were significantly higher in the thalamus than the anterior cingulate cortex; when non‐water‐scaled and non‐*T*_2_‐corrected measurements were reported, Glx/tCr and GSH/tCr ratios were significantly lower in the thalamus compared to the anterior cingulate cortex. Any metabolite measurement based on tCr ratio were heavily driven by the tissue content in different brain regions resulting in a similar influence as reported by Tomiyasu et al.^65^ Of note, most existing algorithms perform relaxation correction using *T*_1_/*T*_2_ relaxation times of white matter, gray matter, and metabolites from average adult population as the correction factors. These factors in fetus and neonates have yet to be measured which may cause biased results if the correction factors are implemented incorrectly.

### Sex‐Specific Differences in Brain Metabolites

In another study, GABA+, Glx, and Cho were reported to be higher in the right frontal lobe in male preterm neonates compared to females.^68^ This discovery suggested that sex differences may play a role in the contribution of ex‐utero brain metabolic concentrations and biologic variability due to infant sex. This should be considered in all early‐life studies using MRS.

Overall, the key metabolites, including NAA and tCr increase while mI and tCho decrease in the fetal‐newborn period. Conflicting results have been reported for GABA and Glx as the modeling of low concentration metabolite peaks are complicated by the low SNR fetal‐neonatal spectra, underlying macromolecule background signals and subtraction artifacts. Further studies are needed to investigate GABA and Glx changes in the developing brain. Similarly, studies to date have revealed regional and sex‐specific differences in metabolite concentrations, all of which should be considered when evaluating early fetal‐neonatal MRS studies. A summary of findings in metabolite concentration changes in healthy term and preterm infants are listed in Table 2. In healthy term and preterm infants without brain injuries, ^1^H‐MRS plays a role in studying neurochemical profiles during the early stage of rapid neurodevelopment. It helps to set a benchmark for the dynamic change of metabolite concentrations during healthy brain maturation.

**TABLE 2:** Brain Metabolite Concentrations in Healthy Term and Preterm Neonates Without Significant Brain Injury

	Preterm (N)	Term (N)	Brain Regions	Field Strength	Vendor	Sequence	Measures	Major Findings
Akasaka et al 2016	0	17	Frontal lobe	1.5 T	Hitachi	PRESS multi‐voxel	/Cr ratio /Cho ratio Longitudinal at 42 PCW and at 3, 6, 9, and 12 months after.	Correlation relationship with PCWs: Positive: NAA/Cr, NAA/Cho Negative: Cho/Cr, Ins/Cr
Basu et al 2019	53	0	Cereb.	1.5 T	GE	PRESS	i.u. /Cr ratio	Correlation relationship with PMAs: Positive: NAA, Cho, and Cr Negative: Cho/Cr
Basu et al 2020	38	0	RFL	3 T	GE	MEGA‐PRESS	i.u.	↑ GABA+, Glx, Cho in male infants compared with female
Basu et al 2022	0	58	Cereb. RBG, RFL	3 T	GE	MEGA‐PRESS	i.u. /Cho ratio	GABA: Cereb. > RBG > RFL Glx: Cereb. > RBG > RFL Cho: Cereb. > RBG > RFL GSH: Cereb. > RBG > RFL NAA: RBG > RFL > Cereb. Cr: RBG > Cereb > RFL GABA+/Cho: RBG > RFL > Cereb. NAA/Cho: RBG > RFL > Cereb. Cr/Cho: RBG > Cereb. > RFL
Basu et al 2023	75	48	Cereb. RBG, RFL	3 T	GE	MEGA‐PRESS	i.u. /Cho ratio	↑ Glx/Cho, GABA+, Cho, GABA+/Cho (adjusted for PMA)
Huppi et al 1991	12	8	Cerebellum	1.5 T	GE	STEAM	mM^a^	↑ NAA, Tau
Koob et al 2016	16	16	CSO	1.5 T	Siemens	PRESS	/H_2_O ratio	↑ Tau, tCr, Glx
Kreis et al 2002	9	16	CSO, thalamus, occipital GM	1.5 T	GE	PRESS	/H_2_O ratio	Metabolite concentrations were significantly different in various brain regions.
Maria et al 2021	0	18	ACC, left thalamus	3 T	Philips	HERMES	i.u. /tCr ratio	Glx/tCr: ACC > Thal. GSH/tCr: ACC > Thal. GABA+: Thal. > ACC Glx: Thal. > ACC
Tanifuji et al 2017	20	20	Right basal ganglia	3 T	GE	PRESS	i.u. /Cr ratio /Cho ratio Longitudinal study	↑ NAA, Glx, Cr, NAA/Cr, Glx/Cr NAA/Cho, Glx/Cho ↓ Ins, GABA/Cr, Cho/Cr, Ins/Cr Ins/Cho (not significant for GABA and GABA/Cho)
Toft et al 1994	9	13	Striatum	1.5 T	Siemens	STEAM	/H_2_O ratio	↑ NAA, PCr + Cr ↓ Inositol
Tomiyasu et al 2013	60	19	BG, CSO, cerebellum	3 T	Siemens	PRESS	mM	↑ tCr, tNAA, Glx, mI (cereb.) ↓ mI (BG) (as a function of PCA)
Tomiyasu et al 2017	28	10	BG, cerebellum	3 T	Siemens	PRESS, MEGA‐PRESS	/H_2_O ratio /tCr ratio	↑ GABA+/H_2_O ↑ Normalized GABA+ (compared with 12 children age of 10.2 years)
Tomiyasu et al 2022	108	0	Deep gray matter, CSO	3 T	Siemens	PRESS	/tCho ratio	↑ tNAA/, tCr/, Glx/tCho in GM ↑ tNAA/tCho in CSO ↓ mI/tCho GM (as a function of PMA)

“↑” Indicates increased metabolite concentrations as a function of age between preterm and term neonates or between neonates and children unless specified for other conditions (vice versa for ↓). “>” Indicates higher metabolite concentrations between the brain regions. ACC = anterior cingulate cortex; Cereb. = cerebellum; CSO = centrum semiovale; GM = gray matter; i.u. = institutional units; LFC = left frontal lobe; PCA = postconceptional week; PMA = postmenstrual age; RBG = right basal ganglia; RFL = right frontal lobe; Thal. = thalamus.

^a^ Using total creatine concentration from human brain autopsy at similar age as an internal endogenous marker.

## MRS in High‐Risk Neonates

Research has investigated the brain's metabolite changes in preterm infants without structural brain injury in comparison to full term healthy newborns. Findings overall are comparable across different studies except for the low concentration metabolites such as GABA and Glx.^52^, ^69^, ^70^, ^71^, ^72^ Discrepancies are most likely in relation to the factors like data quality in the low‐concentrated metabolite, the selection of relevant modeling methods, reporting methods as being “absolute” or “metabolite ratios,” and measures in different brain regions during data acquisition. Apart from healthy neonates, ^1^H‐MRS has been applied to monitor neurochemical changes in sick neonates and their neurodevelopmental outcomes after infancy. The following sections summarize applications of ^1^H‐MRS in neonates with complications or brain diseases.

### Very Low Birth Weight

Preterm neonates at very low birth weight (VLBW) are of specific interest to investigators who study neurochemical profiles using MRS. The NAA/Cho ratio in the preterm VLBW cohort shows significantly decreased in the frontal cortex, hippocampus, and subventricular zone compared to those in healthy controls at term‐equivalent age.^69^ Persistent differences in metabolites (lower Cr, higher Glx/Cr, and higher Cho/Cr) were observed in VLBW preterm children at age 3–4, with related differences in poorer language expression, early executive function, and verbal intelligence quotient (IQ) compared to children born term healthy controls.^71^ Preterm infants small for gestational age due to placental insufficiency were compared to those appropriate‐for‐gestational‐age. However, no significant differences were found.^70^ This study supported the increase of NAA/Cho as a function of age in the gray matter whereas mI/Cho was decreased in both small‐ or appropriate‐for‐gestational‐age groups. A similar study reported a decrease of NAA in preterm with intrauterine growth restriction compared to preterm with healthy weight.^72^ This is of specific interest as preterm and LBW infants are at risk for disrupted brain metabolism with the abrupt cessation of maternal‐placental transfer of key nutrients. ^1^H‐MRS can provide dynamic measures of how these growth delays influence brain development as deceased NAA has been frequently reported which is often used an indicator for healthy brain growth while lower Cr levels may reflect the potential decrease of brain energy metabolism in neonates with VLBW. A more comprehensive summary of clinical data and protocols used for VLBW infants are provided in Table 3.

**TABLE 3:** Brain Metabolite Changes in Very Low Birth Weight (VLBW) Preterm Infants

	VLBW Preterm (N)	Term (N)	Brain Regions	Field Strength	Vendor	Sequence	Measures	Major Findings
Bapat et al 2014	31	12	Subventricular zone, hippocampus, right frontal lobe	3 T	Philips	PRESS	/Cho ratio /mI ratio	↓ NAA/Cho in all three regions‐of‐interest in VLBW infants
Phillips et al 2011^a^	16/12	7/8	ACC, left frontal lobe	3 T	Siemens	PRESS	i.u. /Cr ratio /Cho ratio	↑ Cho/Cr and Glx/Cr and ↓ NAA/Cho and Cr in VLBW children compared to controls at 3–4 years
Roelants‐van Rijn et al 2004b	14/26	0	Basal ganglia	1.5 T	Philips	PRESS	/Cho ratio	No significant difference in metabolite concentrations between AGA and severely SGA infants.
Simoes et al 2017^b^	48	26	Frontal lobe	3 T	Siemens	PRESS	/Cr ratio	↓ NAA in prematurely born intrauterine growth restriction than in prematurely born but adequate for gestational age infants. ↑ NAA in prematurely born but adequate for gestational age compared with term adequate‐for‐gestational‐age infants

ACC = anterior cingulate cortex; AGA = appropriate for gestational age; SGA = small for gestational age.

^a^ Phillips et al (2011) has two preterm groups at different GAs and two corresponding control groups.

^b^ Simoes et al (2017) compared early onset preterm intrauterine growth restriction (P‐IUGR) group with 1) preterm adequate‐for‐gestational‐age (P‐AGA) and 2) term adequate‐for‐gestational‐age (T‐AGA) groups.

### Relationship Between Brain Biochemistry and Cognitive Neurodevelopmental Outcomes

Associations between ^1^H‐MRS brain metabolite concentrations acquired from preterm neonates and their relationship to cognitive outcomes have been reported as well. The most common findings were an association between decreased NAA concentrations to cognitive and neurodevelopment decline and delay regardless of the use of different neuro‐developmental testing. These tests include: the Bayley Scales of Infant and Toddler Development‐III (BSIDIII) scores, Ages and Stages Questionnaire‐2 (ASQ‐2), Differential Abilities Scale (DAS‐II) etc.^69^, ^73^, ^74^, ^75^, ^76^, ^77^ NAA/Cho ratio in the thalamus was reportedly much lower in preterm neonates with a mild developmental delay compared with preterm infants with normal development.^74^ For neonates with low social economic status, there was a negative association reported between Cho/Cr ratio in the left centrum semiovale and motor assessment scores at age 3 years.^78^ The presence of Lac in preterm neonates was associated with lower fine motor and cognitive scores at 18–24 months life.^73^, ^75^ Collectively, these data suggest that ^1^H‐MRS measurements in preterm infants may serve as important biomarkers for cognitive neurodevelopmental outcomes. Clinical data and protocol for association of preterm neonates and cognitive outcomes are listed in Table 4.

**TABLE 4:** Association Between Preterm Infant Brain Biochemistry and Neurodevelopmental Outcomes

	Preterm (N)	Controls (N)	Age at Follow‐Up	Neuro Developmental Test	Brain Regions	Field Strength	Vendor	Sequence	Major Findings
Bapat et al 2014	31	12	18–22 mo.	BSID‐III^a^	Subventricular zone, hippocampus, RFL	3 T	Philips	PRESS	NAA/Cho in subventricular zone and RFL ~ Bayley mental scores
Gire et al 2022	69	N/A	24 mo.	ASQ‐2^a^	FL, PO	1.5 T/3 T	Multiple scanners	PRESS	FL: ↓ NAA/Cho ~ total ASQ and communication score ↑ Lac/Cr motor skills
Hyodo et al 2018	33	16 terms	18 mo.	Kyoto‐Scale	BG	3 T	Siemens	PRESS	↓ NAA/Cho ~ mild developmental delay
Hart et al 2014	33/38	N/A	19 mo.	BSID‐III^a^, Amiel‐Tison	FL/PL	1.5 T	Philips	PRESS	NAA/Cho ~ adverse outcome Lac ~ lower fine motor and lower cognitive composite scores
Van Kooij et al 2012	58	N/A	24 mo.	BSID‐III^a^	Cereb.	3 T	Philips	PRESS	Mother's educational level and NAA/Cho have positive correlation with cognitive scores.
Illapani et al 2022	59	N/A	36 mo.	SES^a^, BSID‐III^a^, DAS‐II^a^	CSO	3 T	Siemens	PRESS	Cho/Cr ~ motor development SES ~ Cho/Cr and motor outcome
Kendall et al 2014	43	N/A	12 mo.	BSID‐III^a^	PL	1.5 T	Siemens	PRESS	NAA/Cho and Cho/Cr ~ cognitive and motor composites

“~” Indicated significant relationship or association between variables. BG = basal ganglia; CSO = centrum semiovale; Cereb. = cerebellum; FL = frontal lobe; mo. = months; PL = posterior lobe; PO = parieto‐occipital; RFL = right frontal lobe; SCI = structural cerebellar injury; WMD = white matter damage.

^a^ Neurodevelopmental tests—ASQ‐2 = Ages and Stages Questionnaire‐2; BSITD‐III = Bayley Scales of Infant and Toddler Development‐III; DAS‐II = Differential Abilities Scale, Second Edition; SES = socioeconomic status.

### Hypertensive Disorders of Pregnancy

Hypertensive disorders of pregnancy (HDP), including chronic hypertension, preeclampsia‐eclampsia, preeclampsia superimposed on chronic hypertension, and gestational hypertension similarly may disrupt the transfer of key metabolites to the developing fetus and result in compromised perinatal transition.^79^ The Research was performed on a group of preterm neonates of HDP mothers. NAA/Cho and NAA/Cr ratio in the bilateral thalami were significantly higher in the HDP group compared with the non‐HDP group. Interestingly, results suggested preterm infants exposed to HDP may experience a utero accelerated brain maturation and increased neuronal activity,^80^ in which relates to one of their previous population‐base studies that suggested preterm infants with HDP have lower odds of adverse neurodevelopmental outcomes compared to the non‐HDP preterm infants.^81^ However, their arguments were solely based on the findings of the altered NAA measurements in the bilateral thalami region. As both preterm birth and HDP can affect levels of brain metabolites, covariances related to preterm birth such as defects or delayed maturation of the respiratory system and comorbidities in the HDP mothers need to be considered.

### Hypoxic–Ischemic Encephalopathy

Hypoxic–ischemic encephalopathy (HIE) at birth is highly associated with long‐term neurodevelopmental impairment due to injury to the developing brain; hence, neonatal patients with HIE have been commonly studied using ^1^H‐MRS to monitor the brain biochemistry at birth and the long‐term neurodevelopment from birth to early childhood. Among the cross‐sectional and serial studies on neonates with HIE within the first week of life, there is a consistent finding of increased Lac, Cho, and Glx, and decreased NAA and Cr compared with healthy controls.^50^, ^82^, ^83^, ^84^, ^85^, ^86^, ^87^, ^88^, ^89^, ^90^, ^91^, ^92^, ^93^, ^94^, ^95^, ^96^, ^97^, ^98^, ^99^, ^100^, ^101^, ^102^, ^103^, ^104^, ^105^ Glycine was occasionally reported in HIE neonates.^86^ Furthermore, early measurements of increased Lac/Cho and Lac/NAA ratios and decreased NAA/Cho ratio were associated with neurodevelopmental status including the neuromotor and cognitive outcomes at age 12 months.^83^ Reduced NAA/Cho ratios were also reported to be associated with reduced fractional anisotropic in diffusion scans in which abnormalities were frequently observed in white matter (91%) and cortex (70%) in neonates with HIE.^106^ It suggested that neonates with HIE suffered brain injuries that adversely affected the brain biochemistry and fiber structure. Persistent Lac detected by ^1^H‐MRS in HIE was associated with alkalosis and an alkaline intracellular pH. The persisting lactic alkalosis was potentially the result of a prolonged effect in the redox state within neuronal cells, presence of phagocytic cells and proliferation of glial cells^107^ and thus a potential marker of anaerobic metabolism that elevated Lac in the HIE brain.

Interestingly, brain temperatures were measured in neonates with encephalopathy due to hypoxic–ischemic using the nature of chemical shift differences detected by ^1^H‐MRS. Most scans were performed during the rewarming process (body temperature >35.5°C) following therapeutic hypothermia, as well as approximately one fifth of the scans were acquired during therapeutic hypothermia or early rewarming.^108^ The brain temperatures were calculated using a formula derived from a phantom calibration or a formula based on chemical shift differences between water and NAA.^109^ Brain temperatures were higher in neonates with more severe HIE than in those with mild HIE. This suggests the potential role of ^1^H‐MRS as a noninvasive method to accurately measure brain temperature during therapeutic hypothermia because maintaining the brain temperature within the therapeutic range for the duration of the therapy is crucial for optimal outcomes of neuroprotection. In a previous randomized clinical trial that studies the efficacy of therapeutic hypothermia, results suggested that a lower temperature and a longer duration of cooling did not change the mortality rate of HIE in NICU.^110^ Of note, core body temperatures were measured and monitored using esophageal probes during cooling to estimate a close approximation of brain temperature. In contrast, MRS utilizes the natural properties of water molecules in the brain to estimate brain temperature based on the chemical shift changes between water and a reference metabolite such as NAA.^109^

### Congenital Heart Disease

Newborns with congenital heart disease (CHD) displayed significantly lower NAA/Cho and higher Lac/Cho ratios under MRS as compared to healthy‐term control infants.^111^, ^112^ White matter NAA/Cho ratios were lower in CHD neonates compared to healthy controls, which might reflect a delay in brain maturation.^113^ In moderate to severe neonatal CHD cases who underwent cardiac surgeries, white matter NAA increased noticeably in postoperative CHD compared to preoperative cases, and the increased level of NAA was comparable to those in healthy controls. However, no correlation was found between pre‐ or postoperative white matter NAA/Cho with the 1‐year neurodevelopment outcome.^114^ The impact of CHD and cardiac surgery on neurodevelopment was uncertain and further longitudinal studies that follow up brain neurochemical on infants with CHD with and without cardiac surgeries may improve our understanding of the clinical impact of altered metabolites.

### Other Systemic Illnesses

^1^H‐MRS is also a tool to investigate cerebral metabolism and has been implemented in the setting of generalized systemic illness, including inborn errors of metabolism, transient metabolic derangements, as well as neonatal hyperbilirubinemia and a myriad of other genetic diseases. Brain metabolite alterations were observed in neonates with severe hyperbilirubinemia (bilirubin levels of ≥25 mg/dL). A high level of Lac/NAA and a low level of NAA/Cho ratios are more possible markers, having been observed in one of the five subjects. However, the statistical power of that study was weak given the small sample size.^115^ A similar study of six neonates with kernicterus reported elevation of Tau/, Glx/, and mI/Cr ratios and a reduction of Cho/Cr ratio compared with normal neonates.^116^ Results from a larger study (N = 31) reported elevated GABA and Cho measurements.^117^ The contradiction of Cho could have been the result of the quantification method being applied as the prior study reported Cho as a ratio of Cr and the later reported absolute concentration. Glucose transporter type 1 deficiency syndrome (GLUT1 DS) is a congenital inborn error of metabolism caused by impaired glucose transports across the blood–brain barrier. A case study reported significantly higher Glx/Cr ratio in the thalamus in GLUT1 DS cases compared to healthy controls. Of note, the comparison was based on two cases and two controls, and it was unclear that they were age‐matched.^118^

Brain metabolite changes have been investigated in neonates with other rare genetic metabolic disorder including isolated sulfite oxidase deficiency,^119^ mitochondrial diseases including respiratory chain defects^120^ and Zellweger Syndrome.^121^, ^122^, ^123^ Common findings included decreased NAA and increased Lac levels, which is similar to the results presented in neonates with brain injuries. In neonates with isolated sulfite oxidase deficiency, ratio of Cho/ and mI/tCr were also increased,^119^ whereas in Zellweger Syndrome, mI level was reduced along with increased glutamine compared to healthy infants.^123^ Inflammatory cytokines could potentially lead to impaired cerebral oxidative metabolism in neonates with encephalopathy resulting in an abnormal neurodevelopmental outcome at 30 months of age.^124^ Results from ^1^H‐MRS suggested that Lac/Cho ratios in the basil ganglia were significantly correlated with various levels of pro‐inflammatory cytokines (IL‐1β, IL‐6, IL‐8, and TNF‐α) but NAA/Cho ratios were not associated with any severity of cytokines in both white and gray matters.^124^ Collectively, these studies suggest that metabolite changes in the brain may be secondary to generalized or somatic metabolic errors but provide new insight into the neurologic manifestations and may provide additional diagnostic and prognostic information to families/providers. Overall, ^1^H‐MRS opens the gate for noninvasive detection of metabolic disorders. Despite more and stronger evidence is needed to support potential biomarkers from the change of brain biochemistry for diagnosis, invasive procedures such as blood tests and spinal taps that are common for testing metabolic disorders could be possibly replaced in the future.

Besides metabolic diseases, neonates with abnormal body conditions such as Chorioamnionitis and Hydrocephalus have been investigated using ^1^H‐MRS. Chorioamnionitis is an infection of the amniotic fluid during pregnancy with a relatively high prevalence in preterm neonates but is uncommon in term neonates.^125^ Researchers studied 31 healthy term neonates with fetal inflammatory response (GA: 39.5 ± 1.3 weeks) and found that decreased NAA/Cho and increased Lac/Cr in basal ganglia were correlated with lower motor and cognitive composite scores, respectively. Developmental outcome at 12‐month showed the increase in NAA/Cho ratios postnatally was slower in infants with below average outcomes.^126^ Hydrocephalus is a condition rather than a disease and is the result of when too much fluid is accumulated in the brain and spinal cord. Absolute brain metabolite concentrations were reported in a small group of term and preterm infants with hydrocephalus. Lactate, glutamine and alanine were reported to be higher compared to the control group.^127^ Another fetal study reported the inositol level to creatine ratio was significantly lower in fetuses with hydrocephalus.^128^

### Effects of Medication

The capacity of ^1^H‐MRS can be extended to study the effect of medical interventions on the neonatal brain. Analgesic medications including opioids may be used on neonates with HIE to ease pain and discomfort during clinical care. Opioid treatment was suggested to have neuroprotective effects as those who treated demonstrated significantly less brain injury during the first week of life.^129^ To further examine the effect of opioid treatment on brain metabolism, 28 asphyxiated term neonates (8 opioid‐treated and 20 non‐opioid) and 6 healthy controls were compared. Decreased NAA/Cr ratio and increased Lac measurements in the occipital gray matter were reported in non–opioid‐treated group compared with the opioid‐treated group.^130^ Neuro‐metabolite observations using ^1^H‐MRS might support the hypothesis that opioids‐treated neonates had less brain injury and received better outcomes.

Interestingly, a study reported Lac/Cho and Lac/NAA ratios were significantly lower in the basal ganglia by 17% and 25% respectively in a group of preterm neonates with pentobarbital sedation compared with the age‐matched neonates without sedation during MRS scans.^131^ This study highlights the necessity of determining medications and sedation for the accurate interpretation of dynamic imaging sequences, such as MRS.

### MRS and Neonatal Prognostic Value

The role of fetal‐neonatal ^1^H‐MRS is not limited to studying the dynamic change of brain metabolites in healthy and high‐risk neonates or correlation between cognitive neurodevelopmental outcomes to metabolites levels. Its application could be further stretched out to predict the outcome or course of a disease. A study evaluated the prognostic value of metabolite ratios using receiver operating characteristics (ROC) analysis. mI/NAA ratio was found to be the best age‐independent predictor for predicting mental developmental outcome of asphyxiated neonates^132^ and Lac/NAA, Lac/Cr, Cho/Cr, tNAA, and myo‐inositol/NAA ratios also showed good prognostic value to predict abnormal or adverse outcomes with high sensitivity and specificity.^111^, ^112^, ^113^, ^133^, ^134^, ^135^, ^136^, ^137^, ^138^, ^139^ NAA/Cho and NAA/Cr ratios in the basal ganglia were found to be significantly correlated with Apgar scores at 5‐minute after born and NAA/Cho ratio with Apgar scores at 1‐minute in a group of term neonates with possible asphyxia.^140^ Lac/NAA ratio was also suggested to be more accurate for early prognosis compared with *T*_2_ relaxation times which positively correlated with developmental outcomes and severity.^137^ Besides hypoxic–ischemic encephalopathy, neuro‐metabolite changes due to trauma, seizures, cardiac arrest and surgery and other miscellaneous reasons were reported in a subgroup of 19 term neonates (mean age at the scan: 11 ± 7.5 days). NAA/Cho, Cho/Cr, NAA/Cr, and NAA measurements were significantly different between neonates with good and poor development outcome. Discriminant analysis suggested clinical variables combined with long echo time data provided up to 95% accuracy on predicting poor outcome on neonates.^141^

## MRS in the Living Fetus

### In Vivo Brain Metabolite Changes in Healthy Fetuses

Fetal ^1^H‐MRS is particularly useful in monitoring and studying neurodevelopmental changes during pregnancy. It is challenging in terms of acquisition, preprocessing, and quantification due to the inherently small volume of the fetus brain and motion artifact, from both the mothers and fetuses, contributing to the low SNR and poor shimming. One of the earliest MRS studies on fetuses demonstrated the feasibility on measuring metabolites at the early stage of the fetal brain.^142^ Longitudinal studies reported the changes of brain metabolite concentrations and results were mostly inline as NAA, Cr, Cho, myo‐inositol, and scyllo‐inositol were increased as a function of age resulting from the rapid development of the fetal brain between the second and the third trimester^143^, ^144^, ^145^ as reported in Table 5. Results also suggested a faster increase in tNAA and tNAA/tCho ratio during the third trimester compared to the second trimester and a faster growth of Cho and tNAA measurements in female subjects.^145^ Some contradicting results were reported as negative correlations were obtained for Cho/Cr ratio in Kok et al^146^ and for mI and Cho in Girard et al.^147^ The discrepancy might be hinted by the rapid changes of the metabolite concentration in the second trimester of the fetus (between 22 and 27 weeks) neurodevelopment and the measuring method for concentration ratio (i.e., sum of all metabolite signal areas as the denominator) contributing to the inconsistency of the findings. Although contradictory results were reported, brain biochemical profile from healthy developing fetal brain set a benchmark for monitoring neurodevelopment of high‐risk fetus during pregnancy. Besides brain development, a recent study demonstrated that elevated maternal depression was associated with decreased Cr and Cho measurements in the fetal brain. This is the first study that has reported on the association between maternal psychological distress and neurobiochemical in fetuses using ^1^H‐MRS.^148^

**TABLE 5:** Brain Metabolites in Healthy and High‐Risk Fetuses

	Healthy Fetuses (N)	Sick Fetuses (N)	Conditions	Brain Regions	Field Strength	Vendor	Sequence	Measures	Major Findings
Andescavage et al 2023	221	112	CHD	Mid. brain	1.5 T	GE	PRESS	i.u. /Cho ratio	↑ Cho ↓NAA/Cho in CHD. Presence of Lac in CHD.
Cetin et al 2011	0	5	IUGR	Mid. brain	1.5 T	Philips	PRESS	i.u.	Presence of Lac in severe cases
Evangelou et al 2016	129	0	Healthy fetuses	Mid. brain	1.5 T	GE	PRESS	i.u. /tCho, /tCr	↑ tNAA, tCho and tCr as function of age
Girard et al 2006	N/A	N/A	GA 22–39 weeks	CSO	1.5 T	N/A	PRESS	/sum of all metabolite signal areas ratio	↑ NAA ↓ mI, Cho between GA 22 and 39 weeks
Kok et al 2001	21	0	Healthy fetuses	Parietal and occipital lobes	1.5 T	Siemens	STEAM	i.u.	^1^H‐MRS in fetal brain is feasible
Kok et al 2002	36	0	Healthy fetuses	Mid. brain (include. scalp and extracranial tissue)	1.5 T	Siemens	STEAM, PRESS	i.u. /Cr ratio	↑ NAA, NAA/Cr, NAA/Cho ↓ Cho/Cr Between GA 30 and 41 weeks
Kok et al 2003	36	10	Hydrocephalus	Mid. brain	1.5 T	Siemens	STEAM	/Cr ratio	↓ Ino/Cr
Limperopoulos et al 2010	55	50	CHD	Centrumovale	1.5 T	Siemens	PRESS	/Cho ratio	↑ NAA/Cho between GA 25 and 37 weeks Lower NAA/Cho progress in CHD compared to controls.
Pradhan et al 2020	112	0	Healthy fetuses	Mid. brain	1.5 T	GE	PRESS	i.u.	↑ tNAA, tCr, tCho, sI, tNAA/tCho with GA. tNAA increased faster in the third trimester. tCho and tNAA increased faster in female
Roelants‐van Rijn et al 2004a	0	6	Hydrocephalus	BG	1.5 T	Philips	PRESS	i.u.	Lac was presented in two cases.
Sanz‐Cortes et al 2015a	55	64	Small for GA	FL	3 T	Siemens	PRESS	i.u. /Cho ratio	↑ Cho/Cr ↓ NAA/Cho in fetuses of small GA
Sanz‐Cortes et al 2015b	30/11^a^	31	Late‐onset IUGR	FL	3 T	Siemens	PRESS	i.u. /Cho ratio	↓ NAA/Cho in SGA and IUGR compared to AGA group
Story et al 2011	41	28	IUGR	Mid. brain	1.5 T	Philips	PRESS	/Cho ratio /Cr ratio	Lac in five IUGR and three fetuses. ↓ NAA/Cr and NAA/Cho in IUGR groups
Story et al 2013	47	28	IUGR	Mid. brain	1.5 T	Philips	PRESS	/Cho ratio /Cr ratio	↓ Cho/Cr, mI/Cho and mI/Cr with advancing GA in control.
Urbanik et al 2019	32	0	Healthy fetuses	Mid. brain	1.5 T	GE	PRESS	i.u.	↑ NAA, Cr, Cho, and mI with advancing GA
Wu et al 2020	119	0	Healthy fetuses	Mid. brain	1.5 T	GE	PRESS	i.u.	NAA, Cr, and Cho

AGA = appropriate for gestational age; BG = basal ganglia; Cereb. = cerebellum; CHD = congenital heart disease; CSO = centrum semiovale; FL = frontal lobe; Hippo = hippocampus; IUGR = intrauterine growth‐restricted; i.u. = institutional unit; SGA = small for gestational age.

^a^ Sanz‐Cortes et al (2015b) recruited 30 fetuses with AGA and 11 with SGA.

### Brain Metabolite Changes in High‐Risk Fetuses

#### CONGENITAL HEART DISEASE

CHD is one of the most common types of cardiovascular birth defects. It increases the risk of abnormal fetal brain development which begins in utero during pregnancies.^149^ Brain metabolites in fetuses with CHD were investigated using ^1^H‐MRS. Data were acquired in a group of fetuses (mean: 31 weeks, range: 25.1–27.1 weeks) with CHD, and those results suggested the rate of increase in NAA/Cho ratio was significantly slower in CHD fetuses when compared to healthy ones.^150^ The abnormal development could be a result of hemodynamic deficiency of blood brain circulation. Another recent study that used a larger cohort of fetuses supported these results. The neurochemical profile was compared between CHD fetuses (N = 170) and healthy controls (N = 333).^151^ Like prior findings, the results showed a decreased NAA/Cho ratio and an increased Cho level in CHD fetuses. Lac peak was reported in all fetuses, which was associated with increased odds of death before discharge. Altered cerebral metabolites in utero during the third trimester hint at cardiac function in fetuses not being capable enough to supply the oxygen that the brain demanded during its rapid developmental stage resulting from hemodynamic deficiency of CHD. The association between CHD and altered brain metabolite changes indicated the differences in brain growth and maturation in the third trimester between CHD and healthy fetuses.

#### FETAL GROWTH RESTRICTION

Brain metabolites in fetuses with fetal growth restriction (FGR) were also reviewed as part of our search. NAA/Cr and NAA/Cho ratios were significantly lower in the FGR groups compared with the healthy controls^152^, ^153^, ^154^; whereas mI/Cho, mI/Cr, and Cho/Cr ratios were not changed between the two groups.^155^ Results were mostly in line with the VLBW preterm neonates previously mentioned. As the level of NAA acted as a surrogate marker of neuronal activity, these results explained the association of a NAA decrease to the development of the fetal brain with growth restriction. Lac is a biomarker of fetal metabolic acidemia and was present in the most severe case of FGR which resulted from increased reliance on anaerobic metabolism. Results were consistent with the high lactic acid concentration observed in umbilical blood.^156^

#### FETAL HYDROCEPHALUS

Decreased levels of the inositol/Cr ratio were observed in fetuses with fetal hydrocephalus compared to the healthy controls. Lac was also presented in spectral of hydrocephalus cases due to the reduced cerebral perfusion from elevated intracranial pressure.^157^ Results suggest that inositol played a role in the development of the neurulation process and in the development of the central nervous system.^128^

## Gaps in Knowledge

As mentioned in the previous sections, MRS data acquisition in the fetal‐neonatal cohorts is more challenging than in adults due to the inherent nature of the subjects and the overall population available for recruitment. For those reasons, questions related to brain metabolites are studied primarily based on adult subjects including (1) the consensus understanding of a brain's biochemistry profile, (2) the physical characteristics, such as *T*_1_/*T*_2_ relaxation times, present in different brain tissues and metabolites, and (3) the influence of macromolecules to LCM. As the easier population to study, most of the recent research being reported on brain metabolite changes are as functions of age using adult cohorts.^158^, ^159^ Metabolite changes as functions of age are anticipated in the fetal‐neonatal stage as the brain is in a period of rapid development. However, there is a lack of existing data to support that claim. Despite the measurement of conventional high‐concentrated metabolites in the fetal‐neonatal brain is possible, it is particularly difficult to study the change of low‐concentrated metabolites as artifacts related to motion, poor shimming, and frequency and phase drifts are more sensitive in fetal‐neonatal scans due to their involuntary movements, small brain size, and physiological instability. For some low‐concentrated metabolites such as GABA and GSH, special spectral editing strategies that utilize known *J*‐coupling relationships are required which generally take a longer period of scan time, which is critical in fetal‐neonatal acquisitions, to compensate for a better SNR and require sophisticated software for modeling. An ongoing large‐scale multicenter study on the developing brain will provide opportunities to study the change of multiple metabolites (including both high and low concentration metabolites) in newborns from the neonatal period to children at the age of 10 using a harmonized and standardized protocol.^160^ *T*_1_/*T*_2_ relaxation times for infants cover only for high concentration metabolites including NAA, Cho, Cr, myo‐inositol, and Glx. Low concentration metabolites such as Asp, Asc, GABA, GSH, Tau, and Lac are not studied in infants as often, and even less time has been dedicated to fetuses and neonates. Correct measurements of all common metabolites at different ages would allow the proper performance of *T*_1_/*T*_2_ relaxation corrections. Changes to the macromolecules have also been reported in the adult data even with contradictory results due to different field strength, TE, quantification, and localization methods.^161^, ^162^ In contrast, macromolecules changes in fetuses and neonates are still unclear and are waiting to be investigated.

## Future Direction

As a noninvasive tool to inform the dynamic, metabolic profiles of the developing brain in healthy and high‐risk conditions, ^1^H‐MRS has a strong potential for future use and refinement. The MRS research community has published consensus papers dedicated to providing recommendations and standardization guidelines on topics and issues including advanced localization methods,^163^ *B*_0_ shimming,^164^ motion,^36^ and processing and quantification.^10^ Along with this emerging technology, standardizing must be addressed to improve MRS as a research tool and transform it into a reliable clinical tool for diagnostic and prognostic purposes. Further refinements to the consensus guidelines are vital for its use in pediatric cases, especially for fetal and neonatal scans, given the unique biologic and technical considerations in these populations. Technical advances, such as real‐time tracking tools for motion, localizations that reduce potential artifacts such as chemical displacements and sequences or AI technologies that shortened the scan time that have been used on adults should be tested and implemented into fetal‐newborn scans. The development of new MRS editing sequences that allow quantification of low concentration metabolites such as GABA and GSH improves the overall understanding of the neurochemical profile. These new sequences can be applied to study neurodevelopment of healthy fetuses and neonates and correlations between metabolite changes and neurological diseases.

## Conclusion

Neuroimaging has a consistent and clear record with ^1^H‐MRS as a tool to document brain metabolite concentrations in health and disease, metabolite profiles as functions of age, physical properties such as *T*_1_/*T*_2_ relaxation times for brain tissues and metabolites, macromolecular concentration, and water concentration in adult cohorts. In neonates and fetuses, ^1^H‐MRS has been used to study the change of brain biochemistry in normal brain maturation and those with adverse conditions or complications such as hypoxic–ischemic encephalopathy and preterm neonates with VLBW. Other applications include long term follow up to study the correlation of neurological diseases or lesions at birth with neurodevelopmental outcomes in childhoods. We have also covered some relatively new and uncommon applications of ^1^H‐MRS in fetal‐neonatal scans throughout the review including the use of ^1^H‐MRS to measure brain temperature, to evaluate prognostic values to predict outcome of a disease or disorder and to study the change of metabolites in different sex and brain regional differences. On the technical side, motion artifacts caused by involuntary movements of fetuses and neonates remains one of the biggest challenges. Future application of real‐time motion trackers can help to reduce the impact. LCM and segmentation can be further improved with proper data and analysis from the newborn cohort. This review paper brings forward valuable insight on the basic principles, applications, challenges, and future direction of ^1^H‐MRS implementation across healthy individual neonates and fetuses and patients with varied conditions or diseases.

## Conflict of Interest

The authors declare no conflicts of interest.

## References

[1] Horska A , Barker PB . Imaging of brain tumors: MR spectroscopy and metabolic imaging. Neuroimaging Clin N Am 2010;20(3):293‐310. 20708548 10.1016/j.nic.2010.04.003 PMC2927327

[2] Vaeggemose M , Schulte RF , Laustsen C . Comprehensive literature review of hyperpolarized Carbon‐13 MRI: The road to clinical application. Metabolites 2021;11(4):219. 10.3390/metabo11040219 PMC8067176 33916803

[3] Harris AD , Saleh MG , Edden RA . Edited (1) H magnetic resonance spectroscopy in vivo: Methods and metabolites. Magn Reson Med 2017;77(4):1377‐1389. 28150876 10.1002/mrm.26619 PMC5352552

[4] van der Graaf M . In vivo magnetic resonance spectroscopy: Basic methodology and clinical applications. Eur Biophys J 2010;39(4):527‐540. 19680645 10.1007/s00249-009-0517-y PMC2841275

[5] de Graaf RA . In vivo NMR spectroscopy – Static aspects. In Vivo NMR Spectroscopy. Hoboken, New Jersey: John Wiley & Sons; 2019. p 43‐128.

[6] Provencher SW . Estimation of metabolite concentrations from localized in vivo proton NMR spectra. Magn Reson Med 1993;30(6):672‐679. 8139448 10.1002/mrm.1910300604

[7] Wilson M , Reynolds G , Kauppinen RA , Arvanitis TN , Peet AC . A constrained least‐squares approach to the automated quantitation of in vivo (1)H magnetic resonance spectroscopy data. Magn Reson Med 2011;65(1):1‐12. 20878762 10.1002/mrm.22579

[8] Clarke WT , Stagg CJ , Jbabdi S . FSL‐MRS: An end‐to‐end spectroscopy analysis package. Magn Reson Med 2021;85(6):2950‐2964. 33280161 10.1002/mrm.28630 PMC7116822

[9] Oeltzschner G , Zollner HJ , Hui SCN , et al. Osprey: Open‐source processing, reconstruction & estimation of magnetic resonance spectroscopy data. J Neurosci Methods 2020;343:108827. 32603810 10.1016/j.jneumeth.2020.108827 PMC7477913

[10] Near J , Harris AD , Juchem C , et al. Preprocessing, analysis and quantification in single‐voxel magnetic resonance spectroscopy: experts' consensus recommendations. NMR Biomed 2021;34(5):e4257. 32084297 10.1002/nbm.4257 PMC7442593

[11] Simpson R , Devenyi GA , Jezzard P , Hennessy TJ , Near J . Advanced processing and simulation of MRS data using the FID appliance (FID‐A) – An open source, MATLAB‐based toolkit. Magn Reson Med 2017;77(1):23‐33. 26715192 10.1002/mrm.26091

[12] Hui SCN , Saleh MG , Zöllner HJ , et al. MRSCloud: A cloud‐based MRS tool for basis set simulation. Magn Reson Med 2022;88(5):1994‐2004. 35775808 10.1002/mrm.29370 PMC9420769

[13] Oz G , Alger JR , Barker PB , et al. Clinical proton MR spectroscopy in central nervous system disorders. Radiology 2014;270(3):658‐679. 24568703 10.1148/radiol.13130531 PMC4263653

[14] Choi C , Ganji SK , DeBerardinis RJ , et al. 2‐hydroxyglutarate detection by magnetic resonance spectroscopy in IDH‐mutated patients with gliomas. Nat Med 2012;18(4):624‐629. 22281806 10.1038/nm.2682 PMC3615719

[15] Branzoli F , Deelchand DK , Sanson M , Lehericy S , Marjanska M . In vivo (1) H MRS detection of cystathionine in human brain tumors. Magn Reson Med 2019;82(4):1259‐1265. 31131476 10.1002/mrm.27810 PMC6626581

[16] Simone V , Rizzo D , Cocciolo A , et al. Infantile brain tumors: A review of literature and future perspectives. Diagnostics (Basel) 2021;11(4):670. 10.3390/diagnostics11040670 PMC8068230 33917833

[17] Bottomley PA . Spatial localization in NMR spectroscopy in vivo. Ann N Y Acad Sci 1987;508:333‐348. 3326459 10.1111/j.1749-6632.1987.tb32915.x

[18] Perdue MV , DeMayo MM , Bell TK , et al. Changes in brain metabolite levels across childhood. Neuroimage 2023;274:120087. 37080345 10.1016/j.neuroimage.2023.120087

[19] Blusztajn JK , Slack BE , Mellott TJ . Neuroprotective actions of dietary choline. Nutrients 2017;9:8. 10.3390/nu9080815 PMC5579609 28788094

[20] Zhu H , Barker PB . MR spectroscopy and spectroscopic imaging of the brain. Methods Mol Biol 2011;711:203‐226. 21279603 10.1007/978-1-61737-992-5_9 PMC3416028

[21] Mescher M , Merkle H , Kirsch J , Garwood M , Gruetter R . Simultaneous in vivo spectral editing and water suppression. NMR Biomed 1998;11(6):266‐272. 9802468 10.1002/(sici)1099-1492(199810)11:6<266::aid-nbm530>3.0.co;2-j

[22] Terpstra M , Marjanska M , Henry PG , Tkac I , Gruetter R . Detection of an antioxidant profile in the human brain in vivo via double editing with MEGA‐PRESS. Magn Reson Med 2006;56(6):1192‐1199. 17089366 10.1002/mrm.21086

[23] Chan KL , Puts NA , Schar M , Barker PB , Edden RA . HERMES: Hadamard encoding and reconstruction of MEGA‐edited spectroscopy. Magn Reson Med 2016;76(1):11‐19. 27089868 10.1002/mrm.26233 PMC5385137

[24] Oeltzschner G , Saleh MG , Rimbault D , et al. Advanced Hadamard‐encoded editing of seven low‐concentration brain metabolites: Principles of HERCULES. Neuroimage 2019;185:181‐190. 30296560 10.1016/j.neuroimage.2018.10.002 PMC6289748

[25] Hui SCN , Murali‐Manohar S , Zöllner HJ , et al. Integrated short‐TE and Hadamard‐edited multi‐sequence (ISTHMUS) for advanced MRS. J Neurosci Methods 2024;409:110206. 38942238 10.1016/j.jneumeth.2024.110206 PMC11286357

[26] Edden RA , Puts NA , Barker PB . Macromolecule‐suppressed GABA‐edited magnetic resonance spectroscopy at 3T. Magn Reson Med 2012;68(3):657‐661. 22777748 10.1002/mrm.24391 PMC3459680

[27] Ben‐Ari Y . Excitatory actions of gaba during development: The nature of the nurture. Nat Rev Neurosci 2002;3(9):728‐739. 12209121 10.1038/nrn920

[28] Chan KL , Oeltzschner G , Saleh MG , Edden RAE , Barker PB . Simultaneous editing of GABA and GSH with Hadamard‐encoded MR spectroscopic imaging. Magn Reson Med 2019;82(1):21‐32. 30793803 10.1002/mrm.27702 PMC6491241

[29] Terpstra M , Gruetter R . ^1^H NMR detection of vitamin C in human brain in vivo. Magn Reson Med 2004;51(2):225‐229. 14755644 10.1002/mrm.10715

[30] Lunsing RJ , Strating K , de Koning TJ , Sijens PE . Diagnostic value of MRS‐quantified brain tissue lactate level in identifying children with mitochondrial disorders. Eur Radiol 2017;27(3):976‐984. 27271921 10.1007/s00330-016-4454-8 PMC5306328

[31] Hui SCN , Zollner HJ , Oeltzschner G , Edden RAE , Saleh MG . In vivo spectral editing of phosphorylethanolamine. Magn Reson Med 2022;87(1):50‐56. 34411324 10.1002/mrm.28976 PMC8616810

[32] Saleh MG , Wang M , Mikkelsen M , et al. Simultaneous edited MRS of GABA, glutathione, and ethanol. NMR Biomed 2020;33:e4227. 31943424 10.1002/nbm.4227 PMC7405912

[33] Cecil KM , Naidu P . Advances in pediatric neuroimaging. MR spectroscopy. Semin Pediatr Neurol 2020;33:100798. 32331612 10.1016/j.spen.2020.100798

[34] Song Y , Lally PJ , Yanez Lopez M , et al. Edited magnetic resonance spectroscopy in the neonatal brain. Neuroradiology 2022;64(2):217‐232. 34654960 10.1007/s00234-021-02821-9 PMC8887832

[35] Kreis R . Issues of spectral quality in clinical ^1^H‐magnetic resonance spectroscopy and a gallery of artifacts. NMR Biomed 2004;17(6):361‐381. 15468083 10.1002/nbm.891

[36] Andronesi OC , Bhattacharyya PK , Bogner W , et al. Motion correction methods for MRS: experts' consensus recommendations. NMR Biomed 2021;34(5):e4364. 33089547 10.1002/nbm.4364 PMC7855523

[37] du Plessis L , Jacobson JL , Jacobson SW , et al. An in vivo ^1^H magnetic resonance spectroscopy study of the deep cerebellar nuclei in children with fetal alcohol spectrum disorders. Alcohol Clin Exp Res 2014;38(5):1330‐1338. 24655149 10.1111/acer.12380 PMC4171650

[38] Mbugua KK , Holmes MJ , Cotton MF , et al. HIV‐associated CD4+/CD8+ depletion in infancy is associated with neurometabolic reductions in the basal ganglia at age 5 years despite early antiretroviral therapy. Aids 2016;30(9):1353‐1362. 26959509 10.1097/QAD.0000000000001082 PMC4864158

[39] Juchem C , de Graaf RA . B(0) magnetic field homogeneity and shimming for in vivo magnetic resonance spectroscopy. Anal Biochem 2017;529:17‐29. 27293215 10.1016/j.ab.2016.06.003 PMC5148734

[40] Branson HM . Normal myelination: A practical pictorial review. Neuroimaging Clin N Am 2013;23(2):183‐195. 23608684 10.1016/j.nic.2012.12.001

[41] Filimonova E , Amelina E , Sazonova A , Zaitsev B , Rzaev J . Assessment of normal myelination in infants and young children using the T1w/T2w mapping technique. Front Neurosci 2023;17:1102691. 36925743 10.3389/fnins.2023.1102691 PMC10011126

[42] Oishi K , Mori S , Donohue PK , et al. Multi‐contrast human neonatal brain atlas: Application to normal neonate development analysis. Neuroimage 2011;56(1):8‐20. 21276861 10.1016/j.neuroimage.2011.01.051 PMC3066278

[43] Makropoulos A , Robinson EC , Schuh A , et al. The developing human connectome project: A minimal processing pipeline for neonatal cortical surface reconstruction. Neuroimage 2018;173:88‐112. 29409960 10.1101/125526 PMC6783314

[44] Harris AD , Puts NA , Edden RA . Tissue correction for GABA‐edited MRS: Considerations of voxel composition, tissue segmentation, and tissue relaxations. J Magn Reson Imaging 2015;42(5):1431‐1440. 26172043 10.1002/jmri.24903 PMC4615266

[45] Kreis R , Ernst T , Ross BD . Development of the human brain: In vivo quantification of metabolite and water content with proton magnetic resonance spectroscopy. Magn Reson Med 1993;30(4):424‐437. 8255190 10.1002/mrm.1910300405

[46] Toft PB , Leth H , Lou HC , Pryds O , Henriksen O . Metabolite concentrations in the developing brain estimated with proton MR spectroscopy. J Magn Reson Imaging 1994;4(5):674‐680. 7981512 10.1002/jmri.1880040510

[47] Kugel H , Roth B , Pillekamp F , et al. Proton spectroscopic metabolite signal relaxation times in preterm infants: A prerequisite for quantitative spectroscopy in infant brain. J Magn Reson Imaging 2003;17(6):634‐640. 12766891 10.1002/jmri.10315

[48] Marjanska M , Emir UE , Deelchand DK , Terpstra M . Faster metabolite (1)H transverse relaxation in the elder human brain. PLoS One 2013;8(10):e77572. 24098589 10.1371/journal.pone.0077572 PMC3788805

[49] Murali‐Manohar S , Gudmundson AT , Hupfeld KE , et al. Metabolite T(1) relaxation times decrease across the adult lifespan. NMR Biomed 2024;37:e5152. 38565525 10.1002/nbm.5152 PMC11303093

[50] Cheong JL , Cady EB , Penrice J , Wyatt JS , Cox IJ , Robertson NJ . Proton MR spectroscopy in neonates with perinatal cerebral hypoxic‐ischemic injury: Metabolite peak‐area ratios, relaxation times, and absolute concentrations. AJNR Am J Neuroradiol 2006;27(7):1546‐1554. 16908578 PMC7977542

[51] Godenschweger F , Kagebein U , Stucht D , et al. Motion correction in MRI of the brain. Phys Med Biol 2016;61(5):R32‐R56. 26864183 10.1088/0031-9155/61/5/R32 PMC4930872

[52] Basu SK , Pradhan S , du Plessis AJ , Ben‐Ari Y , Limperopoulos C . GABA and glutamate in the preterm neonatal brain: In‐vivo measurement by magnetic resonance spectroscopy. Neuroimage 2021;238:118215. 34058332 10.1016/j.neuroimage.2021.118215 PMC8404144

[53] Xu D , Vigneron D . Magnetic resonance spectroscopy imaging of the newborn brain‐‐a technical review. Semin Perinatol 2010;34(1):20‐27. 20109969 10.1053/j.semperi.2009.10.003 PMC2842012

[54] Cecil KM , Jones BV . Magnetic resonance spectroscopy of the pediatric brain. Top Magn Reson Imaging 2001;12(6):435‐452. 11744879 10.1097/00002142-200112000-00005

[55] Liserre R , Pinelli L , Gasparotti R . MR spectroscopy in pediatric neuroradiology. Transl Pediatr 2021;10(4):1169‐1200. 34012861 10.21037/tp-20-445 PMC8107850

[56] Nicola J , Robertson IJC . Magnetic resonance spectroscopy of the neonatal brain. In: Rutherford MA , editor. MRI of the neonatal brain. Philadelphia: W.B. Saunders; 2002.

[57] Huppi PS , Posse S , Lazeyras F , Burri R , Bossi E , Herschkowitz N . Magnetic resonance in preterm and term newborns: ^1^H‐spectroscopy in developing human brain. Pediatr Res 1991;30(6):574‐578. 1666670 10.1203/00006450-199112000-00017

[58] Kreis R , Hofmann L , Kuhlmann B , Boesch C , Bossi E , Huppi PS . Brain metabolite composition during early human brain development as measured by quantitative in vivo ^1^H magnetic resonance spectroscopy. Magn Reson Med 2002;48(6):949‐958. 12465103 10.1002/mrm.10304

[59] Akasaka M , Kamei A , Araya N , et al. Assessing temporal brain metabolite changes in preterm infants using multivoxel magnetic resonance spectroscopy. Magn Reson Med Sci 2016;15(2):187‐192. 26567757 10.2463/mrms.mp.2015-0041 PMC5600055

[60] Basu SK , Pradhan S , Barnett SD , et al. Regional differences in gamma‐aminobutyric acid and glutamate concentrations in the healthy newborn brain. AJNR Am J Neuroradiol 2022;43(1):125‐131. 34764083 10.3174/ajnr.A7336 PMC8757541

[61] Tomiyasu M , Shibasaki J , Kawaguchi H , et al. Altered brain metabolite concentration and delayed neurodevelopment in preterm neonates. Pediatr Res 2022;91(1):197‐203. 33674742 10.1038/s41390-021-01398-6 PMC8770132

[62] Tomiyasu M , Aida N , Endo M , et al. Neonatal brain metabolite concentrations: An in vivo magnetic resonance spectroscopy study with a clinical MR system at 3 Tesla. PLoS One 2013;8(11):e82746. 24312433 10.1371/journal.pone.0082746 PMC3842974

[63] Koob M , Viola A , Le Fur Y , et al. Creatine, glutamine plus glutamate, and macromolecules are decreased in the central white matter of premature neonates around term. PLoS One 2016;11(8):e0160990. 27547969 10.1371/journal.pone.0160990 PMC4993494

[64] Tanifuji S , Akasaka M , Kamei A , et al. Temporal brain metabolite changes in preterm infants with normal development. Brain Dev 2017;39(3):196‐202. 27838187 10.1016/j.braindev.2016.10.006

[65] Tomiyasu M , Aida N , Shibasaki J , et al. In vivo estimation of gamma‐aminobutyric acid levels in the neonatal brain. NMR Biomed 2017;30(1):e3666. 10.1002/nbm.3666 PMC5216898 27859844

[66] Maria YL , Price AN , Puts NAJ , et al. Simultaneous quantification of GABA, Glx and GSH in the neonatal human brain using magnetic resonance spectroscopy. Neuroimage 2021;233:117930. 33711485 10.1016/j.neuroimage.2021.117930 PMC8204265

[67] Basu SK , Pradhan S , Sharker YM , et al. Severity of prematurity and age impact early postnatal development of GABA and glutamate systems. Cereb Cortex 2023;33(12):7386‐7394. 36843135 10.1093/cercor/bhad046 PMC10267637

[68] Basu SK , Pradhan S , Jacobs MB , et al. Age and sex influences gamma‐aminobutyric acid concentrations in the developing brain of very premature infants. Sci Rep 2020;10(1):10549. 32601466 10.1038/s41598-020-67188-y PMC7324587

[69] Bapat R , Narayana PA , Zhou Y , Parikh NA . Magnetic resonance spectroscopy at term‐equivalent age in extremely preterm infants: Association with cognitive and language development. Pediatr Neurol 2014;51(1):53‐59. 24938140 10.1016/j.pediatrneurol.2014.03.011 PMC5942892

[70] Roelants‐van Rijn AM , van der Grond J , Stigter RH , de Vries LS , Groenendaal F . Cerebral structure and metabolism and long‐term outcome in small‐for‐gestational‐age preterm neonates. Pediatr Res 2004;56(2):285‐290. 15181199 10.1203/01.PDR.0000132751.09067.3F

[71] Phillips JP , Ruhl D , Montague E , et al. Anterior cingulate and frontal lobe white matter spectroscopy in early childhood of former very LBW premature infants. Pediatr Res 2011;69(3):224‐229. 21135758 10.1203/PDR.0b013e3182091d52 PMC3107034

[72] Simoes RV , Munoz‐Moreno E , Cruz‐Lemini M , et al. Brain metabolite alterations in infants born preterm with intrauterine growth restriction: Association with structural changes and neurodevelopmental outcome. Am J Obstet Gynecol 2017;216(1):62.e1‐62.e14. 10.1016/j.ajog.2016.09.089 27667762

[73] Gire C , Berbis J , Dequin M , et al. A correlation between magnetic resonance spectroscopy (1‐H MRS) and the neurodevelopment of two‐year‐olds born preterm in an EPIRMEX cohort study. Front Pediatr 2022;10:936130. 36061395 10.3389/fped.2022.936130 PMC9437452

[74] Hyodo R , Sato Y , Ito M , et al. Magnetic resonance spectroscopy in preterm infants: Association with neurodevelopmental outcomes. Arch Dis Child Fetal Neonatal Ed 2018;103(3):F238‐F244. 28724545 10.1136/archdischild-2016-311403

[75] Hart AR , Smith MF , Whitby EH , et al. Diffusion‐weighted imaging and magnetic resonance proton spectroscopy following preterm birth. Clin Radiol 2014;69(8):870‐879. 24935906 10.1016/j.crad.2014.04.001

[76] Van Kooij BJ , Benders MJ , Anbeek P , Van Haastert IC , De Vries LS , Groenendaal F . Cerebellar volume and proton magnetic resonance spectroscopy at term, and neurodevelopment at 2 years of age in preterm infants. Dev Med Child Neurol 2012;54(3):260‐266. 22211363 10.1111/j.1469-8749.2011.04168.x

[77] Kendall GS , Melbourne A , Johnson S , et al. White matter NAA/Cho and Cho/Cr ratios at MR spectroscopy are predictive of motor outcome in preterm infants. Radiology 2014;271(1):230‐238. 24475798 10.1148/radiol.13122679

[78] Illapani VSP , Edmondson DA , Cecil KM , et al. Magnetic resonance spectroscopy brain metabolites at term and 3‐year neurodevelopmental outcomes in very preterm infants. Pediatr Res 2022;92(1):299‐306. 33654289 10.1038/s41390-021-01434-5 PMC8410891

[79] Mammaro A , Carrara S , Cavaliere A , et al. Hypertensive disorders of pregnancy. J Prenat Med 2009;3(1):1‐5. 22439030 PMC3279097

[80] Katsuki S , Ushida T , Kidokoro H , et al. Hypertensive disorders of pregnancy and alterations in brain metabolites in preterm infants: A multi‐voxel proton MR spectroscopy study. Early Hum Dev 2021;163:105479. 34624700 10.1016/j.earlhumdev.2021.105479

[81] Nakamura N , Ushida T , Nakatochi M , et al. Mortality and neurological outcomes in extremely and very preterm infants born to mothers with hypertensive disorders of pregnancy. Sci Rep 2021;11(1):1729. 33462302 10.1038/s41598-021-81292-7 PMC7814115

[82] Groenendaal F , Veenhoven RH , van der Grond J , Jansen GH , Witkamp TD , de Vries LS . Cerebral lactate and N‐acetyl‐aspartate/choline ratios in asphyxiated full‐term neonates demonstrated in vivo using proton magnetic resonance spectroscopy. Pediatr Res 1994;35(2):148‐151. 8165047 10.1203/00006450-199402000-00004

[83] Barkovich AJ , Baranski K , Vigneron D , et al. Proton MR spectroscopy for the evaluation of brain injury in asphyxiated, term neonates. AJNR Am J Neuroradiol 1999;20(8):1399‐1405. 10512219 PMC7657756

[84] Pu Y , Li QF , Zeng CM , et al. Increased detectability of alpha brain glutamate/glutamine in neonatal hypoxic‐ischemic encephalopathy. AJNR Am J Neuroradiol 2000;21(1):203‐212. 10669252 PMC7976324

[85] Roelants‐Van Rijn AM , van der Grond J , de Vries LS , Groenendaal F . Value of (1)H‐MRS using different echo times in neonates with cerebral hypoxia‐ischemia. Pediatr Res 2001;49(3):356‐362. 11228261 10.1203/00006450-200103000-00009

[86] Malik GK , Pandey M , Kumar R , Chawla S , Rathi B , Gupta RK . MR imaging and in vivo proton spectroscopy of the brain in neonates with hypoxic ischemic encephalopathy. Eur J Radiol 2002;43(1):6‐13. 12065114 10.1016/s0720-048x(01)00435-1

[87] Fan G , Wu Z , Chen L , Guo Q , Ye B , Mao J . Hypoxia‐ischemic encephalopathy in full‐term neonate: Correlation proton MR spectroscopy with MR imaging. Eur J Radiol 2003;45(2):91‐98. 12536086 10.1016/s0720-048x(02)00021-9

[88] Kadri M , Shu S , Holshouser B , et al. Proton magnetic resonance spectroscopy improves outcome prediction in perinatal CNS insults. J Perinatol 2003;23(3):181‐185. 12732853 10.1038/sj.jp.7210913

[89] Zhu W , Zhong W , Qi J , Yin P , Wang C , Chang L . Proton magnetic resonance spectroscopy in neonates with hypoxic‐ischemic injury and its prognostic value. Transl Res 2008;152(5):225‐232. 19010293 10.1016/j.trsl.2008.09.004

[90] Alderliesten T , de Vries LS , Staats L , et al. MRI and spectroscopy in (near) term neonates with perinatal asphyxia and therapeutic hypothermia. Arch Dis Child Fetal Neonatal Ed 2017;102(2):F147‐F152. 27553589 10.1136/archdischild-2016-310514

[91] Hanrahan JD , Cox IJ , Azzopardi D , et al. Relation between proton magnetic resonance spectroscopy within 18 hours of birth asphyxia and neurodevelopment at 1 year of age. Dev Med Child Neurol 1999;41(2):76‐82. 10075092 10.1017/s0012162299000171

[92] Hanrahan JD , Sargentoni J , Azzopardi D , et al. Cerebral metabolism within 18 hours of birth asphyxia: A proton magnetic resonance spectroscopy study. Pediatr Res 1996;39(4 Pt 1):584‐590. 8848329 10.1203/00006450-199604000-00004

[93] Kimura H , Fujii Y , Itoh S , et al. Metabolic alterations in the neonate and infant brain during development: Evaluation with proton MR spectroscopy. Radiology 1995;194(2):483‐489. 7529934 10.1148/radiology.194.2.7529934

[94] Hanrahan JD , Cox IJ , Edwards AD , et al. Persistent increases in cerebral lactate concentration after birth asphyxia. Pediatr Res 1998;44(3):304‐311. 9727705 10.1203/00006450-199809000-00007

[95] Penrice J , Cady EB , Lorek A , et al. Proton magnetic resonance spectroscopy of the brain in normal preterm and term infants, and early changes after perinatal hypoxia‐ischemia. Pediatr Res 1996;40(1):6‐14. 8798238 10.1203/00006450-199607000-00002

[96] L'Abee C , de Vries LS , van der Grond J , Groenendaal F . Early diffusion‐weighted MRI and ^1^H‐magnetic resonance spectroscopy in asphyxiated full‐term neonates. Biol Neonate 2005;88(4):306‐312. 16113525 10.1159/000087628

[97] Shibasaki J , Aida N , Morisaki N , Tomiyasu M , Nishi Y , Toyoshima K . Changes in brain metabolite concentrations after neonatal hypoxic‐ischemic encephalopathy. Radiology 2018;288(3):840‐848. 29893645 10.1148/radiol.2018172083

[98] Guo L , Wang D , Bo G , Zhang H , Tao W , Shi Y . Early identification of hypoxic‐ischemic encephalopathy by combination of magnetic resonance (MR) imaging and proton MR spectroscopy. Exp Ther Med 2016;12(5):2835‐2842. 27882082 10.3892/etm.2016.3740 PMC5103703

[99] Robertson NJ , Kuint J , Counsell TJ , et al. Characterization of cerebral white matter damage in preterm infants using ^1^H and ^31^P magnetic resonance spectroscopy. J Cereb Blood Flow Metab 2000;20(10):1446‐1456. 11043907 10.1097/00004647-200010000-00006

[100] Robertson NJ , Lewis RH , Cowan FM , et al. Early increases in brain myo‐inositol measured by proton magnetic resonance spectroscopy in term infants with neonatal encephalopathy. Pediatr Res 2001;50(6):692‐700. 11726726 10.1203/00006450-200112000-00011

[101] Basu SK , Pradhan S , Kapse K , et al. Third trimester cerebellar metabolite concentrations are decreased in very premature infants with structural brain injury. Sci Rep 2019;9(1):1212. 30718546 10.1038/s41598-018-37203-4 PMC6362247

[102] Barkovich AJ , Miller SP , Bartha A , et al. MR imaging, MR spectroscopy, and diffusion tensor imaging of sequential studies in neonates with encephalopathy. AJNR Am J Neuroradiol 2006;27(3):533‐547. 16551990 PMC7976955

[103] Lucke AM , Shetty AN , Hagan JL , et al. Early proton magnetic resonance spectroscopy during and after therapeutic hypothermia in perinatal hypoxic‐ischemic encephalopathy. Pediatr Radiol 2019;49(7):941‐950. 30918993 10.1007/s00247-019-04383-8

[104] Miller SP , Newton N , Ferriero DM , et al. Predictors of 30‐month outcome after perinatal depression: Role of proton MRS and socioeconomic factors. Pediatr Res 2002;52(1):71‐77. 12084850 10.1203/00006450-200207000-00014

[105] Peden CJ , Rutherford MA , Sargentoni J , Cox IJ , Bryant DJ , Dubowitz LM . Proton spectroscopy of the neonatal brain following hypoxic‐ischaemic injury. Dev Med Child Neurol 1993;35(6):502‐510. 8504892 10.1111/j.1469-8749.1993.tb11680.x

[106] Lally PJ , Price DL , Pauliah SS , et al. Neonatal encephalopathic cerebral injury in South India assessed by perinatal magnetic resonance biomarkers and early childhood neurodevelopmental outcome. PLoS One 2014;9(2):e87874. 24505327 10.1371/journal.pone.0087874 PMC3914890

[107] Robertson NJ , Cox IJ , Cowan FM , Counsell SJ , Azzopardi D , Edwards AD . Cerebral intracellular lactic alkalosis persisting months after neonatal encephalopathy measured by magnetic resonance spectroscopy. Pediatr Res 1999;46(3):287‐296. 10473043 10.1203/00006450-199909000-00007

[108] Annink KV , Groenendaal F , Cohen D , et al. Brain temperature of infants with neonatal encephalopathy following perinatal asphyxia calculated using magnetic resonance spectroscopy. Pediatr Res 2020;88(2):279‐284. 31896129 10.1038/s41390-019-0739-3

[109] Wu TW , McLean C , Friedlich P , et al. Brain temperature in neonates with hypoxic‐ischemic encephalopathy during therapeutic hypothermia. J Pediatr 2014;165(6):1129‐1134. 25151196 10.1016/j.jpeds.2014.07.022

[110] Shankaran S , Laptook AR , Pappas A , et al. Effect of depth and duration of cooling on deaths in the NICU among neonates with hypoxic ischemic encephalopathy: A randomized clinical trial. JAMA 2014;312(24):2629‐2639. 25536254 10.1001/jama.2014.16058 PMC4335311

[111] Barta H , Jermendy A , Kovacs L , Schiever N , Rudas G , Szabo M . Predictive performance and metabolite dynamics of proton MR spectroscopy in neonatal hypoxic‐ischemic encephalopathy. Pediatr Res 2022;91(3):581‐589. 34489532 10.1038/s41390-021-01626-z PMC8904256

[112] da Silva LF , Hoefel Filho JR , Anes M , Nunes ML . Prognostic value of ^1^H‐MRS in neonatal encephalopathy. Pediatr Neurol 2006;34(5):360‐366. 16647995 10.1016/j.pediatrneurol.2005.10.011

[113] Holshouser BA , Ashwal S , Luh GY , et al. Proton MR spectroscopy after acute central nervous system injury: Outcome prediction in neonates, infants, and children. Radiology 1997;202(2):487‐496. 9015079 10.1148/radiology.202.2.9015079

[114] Steger C , Feldmann M , Borns J , et al. Neurometabolic changes in neonates with congenital heart defects and their relation to neurodevelopmental outcome. Pediatr Res 2023;93(6):1642‐1650. 35995938 10.1038/s41390-022-02253-y PMC10172141

[115] Groenendaal F , van der Grond J , de Vries LS . Cerebral metabolism in severe neonatal hyperbilirubinemia. Pediatrics 2004;114(1):291‐294. 15231949 10.1542/peds.114.1.291

[116] Oakden WK , Moore AM , Blaser S , Noseworthy MD . ^1^H MR spectroscopic characteristics of kernicterus: A possible metabolic signature. AJNR Am J Neuroradiol 2005;26(6):1571‐1574. 15956531 PMC8149091

[117] Lin Q , Chen L , Zheng H , Tan H , Zhang G , Zheng W . Imaging of nerve injury in neonatal acute bilirubin encephalopathy using (1)H‐MRS and Glu‐CEST techniques. Front Neurosci 2023;17:1110349. 37056307 10.3389/fnins.2023.1110349 PMC10086169

[118] Akasaka M , Kamei A , Araya N , Oyama K , Sasaki M . Characteristic proton magnetic resonance spectroscopy in glucose transporter type 1 deficiency syndrome. Pediatr Int 2018;60(10):978‐979. 30320424 10.1111/ped.13672

[119] Hoffmann C , Ben‐Zeev B , Anikster Y , et al. Magnetic resonance imaging and magnetic resonance spectroscopy in isolated sulfite oxidase deficiency. J Child Neurol 2007;22(10):1214‐1221. 17940249 10.1177/0883073807306260

[120] Dinopoulos A , Cecil KM , Schapiro MB , et al. Brain MRI and proton MRS findings in infants and children with respiratory chain defects. Neuropediatrics 2005;36(5):290‐301. 16217703 10.1055/s-2005-872807

[121] Klouwer FC , Berendse K , Ferdinandusse S , Wanders RJ , Engelen M , Poll‐The BT . Zellweger spectrum disorders: Clinical overview and management approach. Orphanet J Rare Dis 2015;10:151. 26627182 10.1186/s13023-015-0368-9 PMC4666198

[122] Groenendaal F , Bianchi MC , Battini R , et al. Proton magnetic resonance spectroscopy (^1^H‐MRS) of the cerebrum in two young infants with Zellweger syndrome. Neuropediatrics 2001;32(1):23‐27. 11315198 10.1055/s-2001-12218

[123] Bruhn H , Kruse B , Korenke GC , et al. Proton NMR spectroscopy of cerebral metabolic alterations in infantile peroxisomal disorders. J Comput Assist Tomogr 1992;16(3):335‐344. 1592912 10.1097/00004728-199205000-00001

[124] Bartha AI , Foster‐Barber A , Miller SP , et al. Neonatal encephalopathy: Association of cytokines with MR spectroscopy and outcome. Pediatr Res 2004;56(6):960‐966. 15496611 10.1203/01.PDR.0000144819.45689.BB

[125] Jain VG , Kline JE , He L , et al. Acute histologic chorioamnionitis independently and directly increases the risk for brain abnormalities seen on magnetic resonance imaging in very preterm infants. Am J Obstet Gynecol 2022;227(4):623.e1‐623.e13. 10.1016/j.ajog.2022.05.042 PMC10008527 35644247

[126] Johnson CB , Jenkins DD , Bentzley JP , et al. Proton magnetic resonance spectroscopy and outcome in term neonates with chorioamnionitis. J Perinatol 2015;35(12):1030‐1036. 26426253 10.1038/jp.2015.121 PMC4660057

[127] McNatt SA , McComb JG , Nelson MD , Bluml S . Proton magnetic resonance spectroscopy of hydrocephalic infants. Pediatr Neurosurg 2007;43(6):461‐467. 17992033 10.1159/000108788

[128] Kok RD , Steegers‐Theunissen RP , Eskes TK , Heerschap A , van den Berg PP . Decreased relative brain tissue levels of inositol in fetal hydrocephalus. Am J Obstet Gynecol 2003;188(4):978‐980. 12712096 10.1067/mob.2003.206

[129] Angeles DM , Wycliffe N , Michelson D , et al. Use of opioids in asphyxiated term neonates: Effects on neuroimaging and clinical outcome. Pediatr Res 2005;57(6):873‐878. 15774841 10.1203/01.PDR.0000157676.45088.8C

[130] Angeles DM , Ashwal S , Wycliffe ND , et al. Relationship between opioid therapy, tissue‐damaging procedures, and brain metabolites as measured by proton MRS in asphyxiated term neonates. Pediatr Res 2007;61(5 Pt 1):614‐621. 17413864 10.1203/pdr.0b013e318045bde9

[131] Wang ZJ , Vigneron DB , Miller SP , et al. Brain metabolite levels assessed by lactate‐edited MR spectroscopy in premature neonates with and without pentobarbital sedation. AJNR Am J Neuroradiol 2008;29(4):798‐801. 18184837 10.3174/ajnr.A0912 PMC2745552

[132] Barta H , Jermendy A , Kolossvary M , et al. Prognostic value of early, conventional proton magnetic resonance spectroscopy in cooled asphyxiated infants. BMC Pediatr 2018;18(1):302. 30219051 10.1186/s12887-018-1269-6 PMC6139158

[133] Amess PN , Penrice J , Wylezinska M , et al. Early brain proton magnetic resonance spectroscopy and neonatal neurology related to neurodevelopmental outcome at 1 year in term infants after presumed hypoxic‐ischaemic brain injury. Dev Med Child Neurol 1999;41(7):436‐445. 10454226

[134] Khong PL , Tse C , Wong IY , et al. Diffusion‐weighted imaging and proton magnetic resonance spectroscopy in perinatal hypoxic‐ischemic encephalopathy: Association with neuromotor outcome at 18 months of age. J Child Neurol 2004;19(11):872‐881. 15658792 10.1177/08830738040190110501

[135] Shibasaki J , Niwa T , Piedvache A , et al. Comparison of predictive values of magnetic resonance biomarkers based on scan timing in neonatal encephalopathy following therapeutic hypothermia. J Pediatr 2021;239:101‐109.e4. 34391766 10.1016/j.jpeds.2021.08.011

[136] Lally PJ , Montaldo P , Oliveira V , et al. Magnetic resonance spectroscopy assessment of brain injury after moderate hypothermia in neonatal encephalopathy: A prospective multicentre cohort study. Lancet Neurol 2019;18(1):35‐45. 30447969 10.1016/S1474-4422(18)30325-9 PMC6291458

[137] Shanmugalingam S , Thornton JS , Iwata O , et al. Comparative prognostic utilities of early quantitative magnetic resonance imaging spin‐spin relaxometry and proton magnetic resonance spectroscopy in neonatal encephalopathy. Pediatrics 2006;118(4):1467‐1477. 17015537 10.1542/peds.2005-2976

[138] Ancora G , Testa C , Grandi S , et al. Prognostic value of brain proton MR spectroscopy and diffusion tensor imaging in newborns with hypoxic‐ischemic encephalopathy treated by brain cooling. Neuroradiology 2013;55(8):1017‐1025. 23703033 10.1007/s00234-013-1202-5

[139] Zarifi MK , Astrakas LG , Poussaint TY , Plessis AA , Zurakowski D , Tzika AA . Prediction of adverse outcome with cerebral lactate level and apparent diffusion coefficient in infants with perinatal asphyxia. Radiology 2002;225(3):859‐870. 12461272 10.1148/radiol.2253011797

[140] Pavlakis SG , Kingsley PB , Harper R , et al. Correlation of basal ganglia magnetic resonance spectroscopy with Apgar score in perinatal asphyxia. Arch Neurol 1999;56(12):1476‐1481. 10593302 10.1001/archneur.56.12.1476

[141] Holshouser BA , Ashwal S , Shu S , Hinshaw DB Jr . Proton MR spectroscopy in children with acute brain injury: Comparison of short and long echo time acquisitions. J Magn Reson Imaging 2000;11(1):9‐19. 10676615 10.1002/(sici)1522-2586(200001)11:1<9::aid-jmri2>3.0.co;2-6

[142] Kok RD , van den Bergh AJ , Heerschap A , Nijland R , van den Berg PP . Metabolic information from the human fetal brain obtained with proton magnetic resonance spectroscopy. Am J Obstet Gynecol 2001;185(5):1011‐1015. 11717623 10.1067/mob.2001.117677

[143] Urbanik A , Cichocka M , Kozub J , Karcz P , Herman‐Sucharska I . Evaluation of changes in biochemical composition of fetal brain between 18th and 40th gestational week in proton magnetic resonance spectroscopy. J Matern Fetal Neonatal Med 2019;32(15):2493‐2499. 29463154 10.1080/14767058.2018.1439009

[144] Evangelou IE , du Plessis AJ , Vezina G , Noeske R , Limperopoulos C . Elucidating metabolic maturation in the healthy fetal brain using ^1^H‐MR spectroscopy. AJNR Am J Neuroradiol 2016;37(2):360‐366. 26405083 10.3174/ajnr.A4512 PMC7959935

[145] Pradhan S , Kapse K , Jacobs M , et al. Non‐invasive measurement of biochemical profiles in the healthy fetal brain. Neuroimage 2020;219:117016. 32526384 10.1016/j.neuroimage.2020.117016 PMC7491254

[146] Kok RD , van den Berg PP , van den Bergh AJ , Nijland R , Heerschap A . Maturation of the human fetal brain as observed by ^1^H MR spectroscopy. Magn Reson Med 2002;48(4):611‐616. 12353277 10.1002/mrm.10264

[147] Girard N , Fogliarini C , Viola A , et al. MRS of normal and impaired fetal brain development. Eur J Radiol 2006;57(2):217‐225. 16387464 10.1016/j.ejrad.2005.11.021

[148] Wu Y , Lu YC , Jacobs M , et al. Association of prenatal maternal psychological distress with fetal brain growth, metabolism, and cortical maturation. JAMA Netw Open 2020;3(1):e1919940. 31995213 10.1001/jamanetworkopen.2019.19940 PMC6991285

[149] Peyvandi S , Rollins C . Fetal brain development in congenital heart disease. Can J Cardiol 2023;39(2):115‐122. 36174913 10.1016/j.cjca.2022.09.020 PMC9905309

[150] Limperopoulos C , Tworetzky W , McElhinney DB , et al. Brain volume and metabolism in fetuses with congenital heart disease: Evaluation with quantitative magnetic resonance imaging and spectroscopy. Circulation 2010;121(1):26‐33. 20026783 10.1161/CIRCULATIONAHA.109.865568 PMC2819908

[151] Andescavage NN , Pradhan S , Gimovsky AC , et al. Magnetic resonance spectroscopy of brain metabolism in fetuses with congenital heart disease. J Am Coll Cardiol 2023;82(16):1614‐1623. 37821172 10.1016/j.jacc.2023.08.013 PMC13109946

[152] Story L , Damodaram MS , Allsop JM , et al. Brain metabolism in fetal intrauterine growth restriction: A proton magnetic resonance spectroscopy study. Am J Obstet Gynecol 2011;205(5):483.e1–8. 10.1016/j.ajog.2011.06.032 21861969

[153] Sanz‐Cortes M , Egana‐Ugrinovic G , Simoes RV , Vazquez L , Bargallo N , Gratacos E . Association of brain metabolism with sulcation and corpus callosum development assessed by MRI in late‐onset small fetuses. Am J Obstet Gynecol 2015;212(6):804.e1–8. 10.1016/j.ajog.2015.01.041 25640049

[154] Sanz‐Cortes M , Simoes RV , Bargallo N , Masoller N , Figueras F , Gratacos E . Proton magnetic resonance spectroscopy assessment of fetal brain metabolism in late‐onset “small for gestational age” versus “intrauterine growth restriction” fetuses. Fetal Diagn Ther 2015;37(2):108‐116. 25115414 10.1159/000365102

[155] Story L , Damodaram MS , Supramaniam V , et al. Myo‐inositol metabolism in appropriately grown and growth‐restricted fetuses: A proton magnetic resonance spectroscopy study. Eur J Obstet Gynecol Reprod Biol 2013;170(1):77‐81. 23810059 10.1016/j.ejogrb.2013.05.006

[156] Cetin I , Barberis B , Brusati V , et al. Lactate detection in the brain of growth‐restricted fetuses with magnetic resonance spectroscopy. Am J Obstet Gynecol 2011;205(4):350.e1–7. 10.1016/j.ajog.2011.06.020 21861968

[157] Roelants‐van Rijn AM , Groenendaal F , Stoutenbeek P , van der Grond J . Lactate in the foetal brain: Detection and implications. Acta Paediatr 2004;93(7):937‐940. 15303809

[158] Gong T , Hui SCN , Zöllner HJ , et al. Neurometabolic timecourse of healthy aging. Neuroimage 2022;264:119740. 36356822 10.1016/j.neuroimage.2022.119740 PMC9902072

[159] Marjanska M , McCarten JR , Hodges J , et al. Region‐specific aging of the human brain as evidenced by neurochemical profiles measured noninvasively in the posterior cingulate cortex and the occipital lobe using (1)H magnetic resonance spectroscopy at 7 T. Neuroscience 2017;354:168‐177. 28476320 10.1016/j.neuroscience.2017.04.035 PMC5516630

[160] Dean DC 3rd , Tisdall MD , Wisnowski JL , et al. Quantifying brain development in the HEALthy Brain and Child Development (HBCD) study: The magnetic resonance imaging and spectroscopy protocol. Dev Cogn Neurosci 2024;70:101452. 39341120 10.1016/j.dcn.2024.101452 PMC11466640

[161] Hui SCN , Gong T , Zöllner HJ , et al. The macromolecular MR spectrum does not change with healthy aging. Magn Reson Med 2022;87(4):1711‐1719. 34841564 10.1002/mrm.29093 PMC8935352

[162] Genovese G , Terpstra M , Filip P , et al. Age‐related differences in macromolecular resonances observed in ultra‐short‐TE STEAM MR spectra at 7T. Magn Reson Med 2024;92:4‐14. 38441257 10.1002/mrm.30061 PMC11055657

[163] Oz G , Deelchand DK , Wijnen JP , et al. Advanced single voxel (1) H magnetic resonance spectroscopy techniques in humans: Experts' consensus recommendations. NMR Biomed 2020;34:e4236. 10.1002/nbm.4236 PMC7347431 31922301

[164] Juchem C , Cudalbu C , de Graaf RA , et al. B(0) shimming for in vivo magnetic resonance spectroscopy: Experts' consensus recommendations. NMR Biomed 2021;34(5):e4350. 32596978 10.1002/nbm.4350

